# A dynamical model of drinking and smoking with optimal control analysis

**DOI:** 10.1371/journal.pone.0311835

**Published:** 2024-12-19

**Authors:** Fredrick Asenso Wireko, Sebastian Ndogum, Botchew Abdul Nasiru, David Ansah-Asamoah, Isaac Kwasi Adu, Joshua Kiddy K. Asamoah

**Affiliations:** 1 Department of Mathematics, Saveetha School of Engineering SIMATS, Chennai, India; 2 Department of Mathematics, Kwame Nkrumah University of Science and Technology, Kumasi, Ghana; 3 Department of Mathematical Science, Kumasi Technical University, Kumasi, Ashanti Region, Ghana; SRM Institute of Science and Technology (Deemed to be University), INDIA

## Abstract

The intake of alcohol is dangerous, and the smoking of tobacco is savage, but it is life-threatening to practice both smoking and drinking. According to the World Health Organisation, the world loses about 8.5 million people each year as a result of smoking tobacco and drinking alcohol. To study this, we present a mathematical model that investigates the co-dynamics of alcohol drinking and tobacco smoking, as well as some control strategies. In contrast, many studies focus solely on the dynamics of alcohol consumption or tobacco smoking. Also, these studies assume that an individual who may recover from both alcohol drinking and tobacco smoking may relapse. We determined the basic reproductive number by employing the next-generation matrix approach. We conducted local and global stability analyses for the drinking, smoking-free, and endemic states. We then conducted extensive research into secondary infections related to smoking and drinking. We then performed numerical simulations and analysis using the parameter values from the literature. The study further examined the influence of some key parameters on secondary co-dependence infections, which occur when one infected individual enters the population and recovers from both over time. For example, in this study, it was shown that the contact rates *a*_1_ and *a*_2_ have a direct relationship to the spread of drinking and smoking. In contrast, recovery rates *δ*_1_, *δ*_2_ showed an inverse relationship. In addition, we conducted an optimal control analysis by suggesting the following: drinking prevention efforts, smoking prevention efforts, recovery efforts on the co-dependence of drinking and smoking, recovery efforts on drinking, and recovery efforts on smoking. The simulations indicated that using these controls can help reduce the number of smokers and drinkers within eight weeks.

## 1. Introduction

While we typically classify drinking and smoking as complementary products, they have detrimental impacts on people’s health. Unequivocally, their combined effect exacerbates the negative consequences [1]. Alcohol consumption and tobacco smoking are two commonly co-occurring social behaviours, and the combined consumption of these substances is associated with heightened health risks and significant health implications for individuals’ body organs. The dual use of alcohol and tobacco has been found to heighten the risk of various diseases, including cardiovascular diseases, respiratory diseases, and different types of cancer [2]. Studies have shown that alcohol consumption leads to a higher mortality rate than diseases like tuberculosis, HIV/AIDS, and diabetes. An estimated 2.3 million deaths and 106.5 million disability-adjusted life years were the result of alcohol consumption in 2016 alone [3]. Tobacco, on the other hand, kills up to half of its users. Researchers estimate that smoking kills over 8 million people annually, including 1.3 million non-smokers exposed to second-hand smoke [4, 5]. Therefore, researchers worldwide are examining ways to control this socially unacceptable behavior. For example, in [6], a study is conducted into fluctuations in ventilation rate and smoking resistance. A recent study by [7] investigated into some neurological dysfunctions that may occur when individuals in the communities become addicted to alcohol usage. After sampling thirty-one patients with alcohol use disorder (AUD) and also thirty-one AUD with control in order to examine their memory functioning, they reported that AUD patients interest in engaging in alcohol usage reduces drastically as neurological factors like anxiety, depression, and impulsiveness are put under control. This explicitly indicates that working memory has an enormous influence on AUD time perception.

The COVID-19 pandemic has contributed immensely to alcohol and tobacco use. A report by [8] indicated that alcohol consumption and tobacco smoking are very prevalent, with 9.3 percent and 14.2 percent, respectively. These rates during the COVID-19 pandemic increased to 20 percent and 30 percent for alcohol consumption and smoking, respectively. These figures were even higher among individuals with depressive feelings, sadness, and stress. The prevalence of the co-dynamics of drinking and smoking varies across different populations and regions. Epidemiological studies have consistently demonstrated a high rate of dual use. For instance, a survey by [9] reported that approximately 80 percent of individuals who smoke also consume alcohol. Moreover, research indicates that the consumption of alcohol and tobacco products is more prevalent among certain demographic groups, such as young adults, males, and individuals with lower socioeconomic status [10].

The use of alcohol and tobacco leads to compounded physiological effects on the body. Alcohol and tobacco interact synergistically, resulting in increased health risks. Alcohol consumption can enhance the harmful effects of smoking, such as oxidative stress, DNA damage, and inflammation [11]. Additionally, individuals who engage in the dual use of alcohol and smoking often experience overlapping psychological effects and addictive behaviors. The dual use of these substances can reinforce each other, leading to increased cravings and sometimes making it challenging to quit both [12]. The combined use of alcohol and smoking is associated with a range of detrimental health outcomes, which significantly elevates the risk of developing chronic diseases, including liver cirrhosis, chronic obstructive pulmonary disease (COPD), and certain types of cancer [13]. Alcohol drinking and smoking are influenced by various sociocultural factors, cultural norms, social environments, and marketing strategies, which play significant roles in shaping the concurrent use of these substances. Societal acceptance of alcohol and tobacco, peer influence, and targeted advertising campaigns also contribute to the dual use of alcohol and smoking [14].

According to the survey reported in [9], it is prudent to study the co-dependence between alcohol and tobacco. Therefore, in this paper, we seek to investigate the dynamics observed when an individual co-depends on alcohol and tobacco. Thus, the study proposes an optimal control model for the co-dependence of drinking and smoking, which incorporates the possibility of an individual quitting both alcohol and tobacco over time, as well as the possibility of relapse after recovery or vulnerability.

We categorize the remainder of the paper into seven sections. Section 2 conducts a comprehensive literature review on smoking and drinking. Section 3 formulates the model using non-linear ordinary differential equations, while section 4 conducts an extensive qualitative analysis. Section 5 investigates the influence of the model’s parameters through a sensitivity index analysis. Section 6 performs an optimal control analysis, suggesting essential and practical strategies to control this social menace. In Section 7, we perform numerical computations and conduct several numerical simulations to assess the impact of critical parameters on the rise in smoking and drinking. Finally, Section 8 concludes the work and highlights future research on this societal problem.

### 1.1. Literature review

Many researchers use mathematical modeling, also known as the modeling of physical phenomena through ordinary differential equations, as a scientific method to study physical problems, their dynamics, and control mechanisms.

Recently, several studies have explored the application of mathematical modelling in the study of smoking and drinking dynamics in society and their consequential effects [15–18]. As previously stated, numerous individuals encounter health issues as a result of engaging in unhealthy lifestyles and behaviors. Even society faces some abnormalities when individuals in the community put up with these unacceptable behaviors. Researchers have conducted scientific studies using mathematical models to understand and control the dynamics of such society-breaking issues.

Through mathematical modelling, many researchers have investigated alcohol and tobacco consumption, and their reports convey fascinating remarks. To name a few, in the study [19], the dynamics of smoking were described by categorizing the population into four compartments. They then conducted a deterministic and stochastic stability analysis of the model. Their study revealed that the extent of perturbations in the model’s parameter values entirely determines the stability of the smoking-present equilibrium. In addition, [16] also developed a mathematical model to study the dynamics of smoking by considering the effects of media information and awareness. In their work, they also took into account the possibility of relapse after recovery. They strongly suggested that media communication is an essential means to create awareness of the adverse effects of smoking, and it stands tall as a significant means to control tobacco smoking.

A study by [20] also presented a mathematical model of the worldwide dynamics of smoking. This model partitions the overall population into three distinct categories: potential smokers (P), current smokers (S), and individuals who have permanently stopped smoking (R). Additionally, the model incorporates the influence of media campaigns (M). The researchers investigated the stability of disease-free and endemic equilibrium points at local and global scales, employing the basic reproductive number as a critical metric. Their findings indicated that increased education has a positive impact on influencing potential smokers to stop smoking, resulting in a decrease in the smoker population. Another study in [21] explores the effects of the smoking epidemic on two age groups, demonstrating the effectiveness of age-targeted interventions in reducing smoking rates.

Recently, [22] carried out a dynamical analysis of alcohol consumption using fractional operators. They validated the model’s existence, uniqueness, and stability through the fixed-point theory and the Hyers-Ulam stability criterion. By performing diverse scenarios for different fractional values, they highlighted how fractional operators could describe the alcoholic epidemic. A study conducted in [23] presented an integer-order model of the drinking epidemic. From their numerical analysis, it was reported that to be able to control this epidemic, there is a need to minimise the interactions between susceptible and heavy drinkers, encourage drinkers to be treated, and also enhance education strategies. Again, [24] developed a mathematical model to examine the dynamics of alcohol consumption, considering the presence of alcohol treatment centres. The researchers categorized the overall population into six distinct compartments, namely potential drinkers P(t), moderate drinkers *M*(*t*), heavy drinkers *H*(*t*), rich heavy drinkers *T*^*r*^(*t*), poor heavy drinkers *T*^*p*^(*t*), and individuals who have ceased drinking, referred to as quitters of drinking *Q*(*t*). They concluded that the local stability of drinking-free equilibrium is asymptotically stable if the basic reproductive number is less than one, and alcohol-present equilibrium is locally asymptotically stable if $R_{0}>1$. Some other significant studies that further proposed mathematical models to efficiently study the dynamics of smoking and drinking in order to enhance sustainable development could be seen in [25–28].

Optimal control is one of the most effective ways of controlling an epidemiological disease or a social canker that causes serious health complications for individuals, and this has received diverse applications in mathematical models to curb the spread of an epidemic disease or a social life canker [29–31]. For instance, in [32], an extensive study on the significance of optimal control was conducted where the mathematical model developed incorporated a smoking model’s passive and active dynamics. Their studies observed that after applying all the suggested controls, the number of passive or active smokers declined drastically. In contrast, an improvement was made in the number of potential smokers and temporary quitters. Optimal control has widely received applications in the studies of nonlinear differential equations; see, for instance, [33, 34].

### 1.2. Research gap

After carefully reviewing the existing literature on tobacco smoking and alcohol drinking we observed that there is not much literature on the use of mathematical modelling to study the co-dependence of alcohol consumption and smoking. The only mathematical model on the co-dynamics of drinking and smoking is [35], which conducted a theoretical analysis of alcoholism and smoking. Their work divided the population into eight compartments, where the local and endemic equilibrium points were studied using the basic reproductive number. Their studies posited that, in a smoking community, there is a greater probability of many people engaging in alcohol consumption than in communities where no one smokes. A similar deduction is made as there is a greater number of individuals smoking among alcohol-consumption communities than in non-alcohol-consumption populations. Hence, drinking and smoking fuel each other. However, they did not consider the fact that an individual can quit both drinking and smoking over time and could even relapse after recovery or become vulnerable again. In their model, a recovered individual can only relapse into either being an addicted drinker or an addicted smoker.

Therefore, in this paper, we seek to develop an optimal control model that studies the co-dependence of drinking and smoking by incorporating in the model that an individual can quit both drinking and smoking over time and may also relapse after recovery or become vulnerable. We assume in this current work that an individual can quit both drinking and smoking simultaneously within a period since abstaining from one will elevate the rehabilitation process in quitting the other; see the references [36, 37]. Moreover, we suggest that an individual who has quit either smoking or drinking or both can only become a drinker or smoker after going through the susceptible compartment. In summary, this current study focuses on:

1. formulating a mathematical model for the co-dependence on drinking and smoking.
2. conducting an optimal control analysis on the co-dependence on drinking and smoking.
3. analysing the dynamics of relapse in the drinking and smoking model.

The co-dependence model for drinking and smoking is formulated in the next section, and further discussions are performed appropriately.

## 2. Formulation of the model

**Fig 1:**
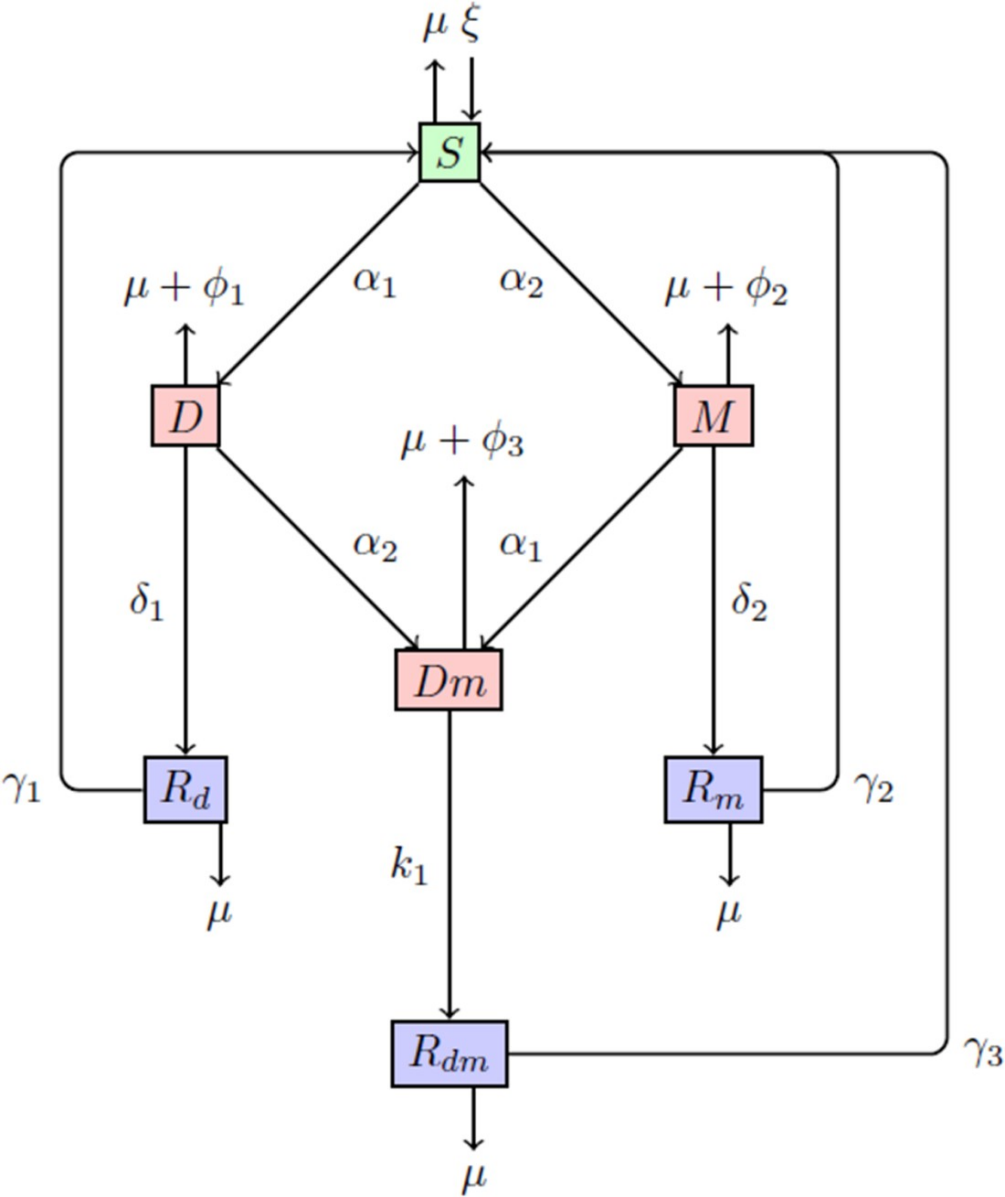
Flow diagram of the model.

In this section, we develop a mathematical model to study the co-dynamics of drinking and smoking. The total population over time *N*(*t*) is classified into seven sub-compartments with continuous interaction among individuals in the population. The compartments consist of the susceptible, denoted by *S*(*t*), individuals in the population that drink alcohol, denoted by *D*(*t*), and individuals in the population that smoke, represented by *M*(*t*). Also, individuals that co-depend on both drinking and smoking are denoted by *Dm*(*t*), and the others are recovered drinker individuals *R*_*d*_(*t*), recovered smoker individuals *R*_*m*_(*t*), and recovered co-dependence individuals *R*_*dm*_(*t*). The recruitment of individuals into the susceptible population is either from birth or peer influence at the rate of *ξ*, and this gets increased as a result of relapse from the recovered drinkers at the rate of *γ*_1_, the recovered smokers at the rate of *γ*_2_, and the recovered drinkers and smokers at the rate of *γ*_3_. Through interactions with peers or advertisements, individuals become drinkers at the rate of *α*_1_ and with a force of co-dependence of drinkers at *α*_1_ = *a*_1_(*D* + *Dm*), where *a*_1_ is the interaction rate of drinkers, thereby increasing the drinker population. Similarly, individuals join the smoking population at the rate of *α*_2_ and, with a force of co-dependence, individuals of smokers at *α*_2_ = *a*_2_(*M* + *Dm*), where *a*_2_ is the contact rate of smokers. Individuals recover from drinking at the rate of *δ*_1_ and move into the *R*_*d*_(*t*) compartment with a drinking-induced death rate of *ϕ*_1_. Individuals also recover from smoking at the rate of *δ*_2_ and move into the *R*_*m*_(*t*) compartment with a smoking-induced death rate of *ϕ*_2_. Individuals recover from both drinking and smoking *Dm*(*t*) overtime at the rate of *k*_1_ and move into the *R*_*dm*_ compartment with a drinking and smoking-induced death rate of *ϕ*_3_. The natural death rate *μ* is considered constant in all compartments. Fig 1 shows the interactions among the sub-classes, and Table 1 describes the parameters used in Fig 1. Some other assumptions necessary for the co-dynamics of the smoking and drinking model are given below;

1. Individuals who smoke could easily become alcohol addicts, and drinkers could also become smokers, as supported in the literature, see [9, 35, 38].
2. There is a temporal recovery; that is, individuals who quit both or either drinking or smoking may become susceptible again [39].
3. Drinking-induced death and smoking-induced death occur at different rates and the co-dependence-induced death rate is the sum of the individual rates, that is *ϕ*_3_ = *ϕ*_1_ + *ϕ*_2_ [35].
4. Individuals can recover from both drinking and smoking over time through rehabilitation, as supported by literature [36, 37].

**Table 1:** Model parameters and their description.

Parameter	Description
*ξ*	Recruitment rate
*a*_1_	Rate at which the susceptible become drinkers only
*a*_2_	Rate at which the susceptible become smokers only
*δ*_1_	Recovering rate of drinkers
*δ*_2_	Recovering rate of smokers
*k*_1_	Recovering rate of both drinkers and smokers
*μ*	Natural death rate
*ϕ*_1_	Alcohol drinkers induced death
*ϕ*_2_	Smokers induced death
*ϕ*_3_	Alcohol drinkers and smokers induced death
*γ*_1_	Transmission rate from *R*_*d*_ to *S*
*γ*_2_	Transmission rate from *R*_*m*_ to *S*
*γ*_3_	Transmission rate from *R*_*dm*_ to *S*

From the model above, we derive the following integer-order ordinary differential equations,

$$\begin{array}{c} \begin{array}{cc} & \frac{dS}{dt}=\xi +\gamma_{1}R_{d}+\gamma_{2}R_{m}+\gamma_{3}R_{dm}-\left(\alpha_{1}+\alpha_{2}+\mu \right)S,\\ & \frac{dD}{dt}=\alpha_{1}S-\left(\mu +\phi_{1}+\delta_{1}+\alpha_{2}\right)D,\\ & \frac{dM}{dt}=\alpha_{2}S-\left(\mu +\phi_{2}+\delta_{2}+\alpha_{1}\right)M,\\ & \frac{dDm}{dt}=\alpha_{2}D+\alpha_{1}M\left(t\right)-\left(\mu +\phi_{3}+k_{1}\right)Dm,\\ & \frac{dR_{D}}{dt}=\delta_{1}D-\left(\mu +\gamma_{1}\right)R_{d},\\ & \frac{dR_{M}}{dt}=\delta_{2}M-\left(\mu +\gamma_{2}\right)R_{m},\\ & \frac{dR_{dm}}{dt}=k_{1}Dm-\left(\mu +\gamma_{3}\right)R_{dm}. \end{array}\} \end{array}$$ (1)

With initial conditions *S*(0) ≥ 0, *D*(0) ≥ 0, *M*(0) ≥ 0, *R*_*d*_(0) ≥ 0, *Dm*(0) ≥ 0, *R*_*m*_(0) ≥ 0, *R*_*dm*_(0)≥0.

## 3. Model analysis

This section presents an essential mathematical analysis that makes the model formidable for the physical environment. We have, therefore, carried out positivity and boundedness analyses to show that the model has non-negative solutions that are also bounded. Further investigations are being carried out to validate that the model is stable.

### 3.1. Invariant regions

To describe model (1) as physically feasible, that is, to demonstrate that the solution exists within a limited region, the total population, denoted by N(t), is stated as:

$$N\left(t\right)=S\left(t\right)+D\left(t\right)+M\left(t\right)+R_{d}\left(t\right)+D_{m}\left(t\right)+R_{m}\left(t\right)+R_{dm}\left(t\right).$$

By differentiating N(t) concerning t and making substitutions from (1), that is,

$$\begin{array}{c} \begin{array}{cc} \frac{dN(t)}{dt} & =\frac{dS(t)}{dt}+\frac{dD(t)}{dt}+\frac{dM(t)}{dt}+\frac{dDm(t)}{dt}+\frac{dR_{d}}{dt}+\frac{dR_{m}}{dt}+\frac{dR_{dm}}{dt},\\ & =\xi +\gamma_{1}R_{d}+\gamma_{2}R_{m}+\gamma_{3}R_{dm}-\left(\alpha_{1}+\alpha_{2}+\mu \right)S\\ & +\alpha_{1}S-\left(\mu +\phi_{1}+\delta_{1}+\alpha_{2}\right)D+\alpha_{2}S-\left(\mu +\phi_{2}+\delta_{2}+\alpha_{1}\right)M\\ & +\alpha_{2}D+\alpha_{1}M-\left(\mu +\phi_{3}+k_{1}\right)Dm+\delta_{1}D-\left(\mu +\gamma_{1}\right)R_{d}\\ & +\delta_{2}M-\left(\mu +\gamma_{2}\right)R_{m}+k_{1}Dm-\left(\mu +\gamma_{3}\right)R_{dm}, \end{array} \end{array}$$

canceling out terms and regrouping, we have:

$$\frac{dN}{dt}=\xi -\mu \left(S+D+M+R_{d}+Dm+R_{m}+R_{dm}\right)-\phi_{3}\left(D+M+Dm\right),$$

but we know that *S* + *D* + *M* + *R*_*d*_ + *Dm* + *R*_*m*_ + *R*_*dm*_ = *N*, hence

$$\frac{dN}{dt}=\xi -\mu N-\phi_{3}\left(D+M+Dm\right),$$

but if *ϕ*_3_ = 0, that is, if there is no death from drinking and smoking-related then,

$$\frac{dN}{dt}\leq \xi -\mu N,$$

$$\frac{dN}{dt}+\mu N\leq \xi .$$

Using the method of integrating factor, we compute N as follows,

$$Ne^{ut}\leq \int \xi e^{ut},$$

$$\Rightarrow Ne^{\mu t}\leq \frac{\xi e^{\mu t}}{\mu },$$

$$\Rightarrow N\leq \frac{\xi }{\mu }.$$

Therefore the solutions are bounded in the region,

$$\Omega =\{\left(S,D,M,R_{d},Dm,R_{m},R_{dm}\right)\in R^{7}:0\leq N\leq \frac{\xi }{\mu }\}.$$

### 3.2. Positivity of solution

**Theorem 3.1**. *If* $S_{0}>0,D_{0}>0,M_{0}>0,Dm_{0}>0,Rd_{0}>0,Rm_{0}>0,R_{dm_{0}}>0,$ *then the solutions* (*S*(*t*), *D*(*t*), *M*(*t*), *Dm*(*t*), *R*_*d*_(*t*), *R*_*m*_(*t*), *R*_*dm*_(*t*)) *are positive as time approaches infinity*.

*Proof*. From Eq (1) above, using the first equation

$$\frac{dS}{dt}=\xi +\gamma_{1}R_{d}+\gamma_{2}R_{m}+\gamma_{3}R_{dm}-\left(\alpha_{1}+\alpha_{2}+\mu \right)S,$$

$$\begin{array}{c} \frac{dS}{dt}+\left(\alpha_{1}+\alpha_{2}+\mu \right)S=\xi +\gamma_{1}R_{d}+\gamma_{2}R_{m}+\gamma_{3}R_{dm}, \end{array}$$ (2)

by using the method of variation of parameters on Eq (2), let us take *S*(*t*) = *u*(*t*)*v*(*t*), where

$$v\left(t\right)=e^{-\int_{0}^{t}\left(\alpha_{1}+\alpha_{2}+\mu \right)dt}$$

and

$$u\left(t\right)=\frac{\left(\xi +\gamma_{1}R_{d}\left(t\right)+\gamma_{2}R_{m}\left(t\right)+\gamma_{3}R_{dm}\left(t\right)\right)}{e^{-\int_{0}^{t}\left(\alpha_{1}+\alpha_{2}+\mu \right)dt}}dt,$$

$$u\left(t\right)=\int_{0}^{t}\left(\xi +\gamma_{1}R_{d}\left(t\right)+\gamma_{2}R_{m}\left(t\right)+\gamma_{3}R_{dm}\left(t\right)\right)e^{\int_{0}^{t}\left(\alpha_{1}+\alpha_{2}+\mu \right)dt}dt,$$

$$\Rightarrow S\left(t\right)=e^{\left(-\int_{0}^{t}\left(\alpha_{1}+\alpha_{2}+\mu \right)dt\right)}\left[\int_{0}^{t}\left(\xi +\gamma_{1}R_{d}\left(t\right)+\gamma_{2}R_{m}\left(t\right)+\gamma_{3}R_{dm}\left(t\right)\right)e^{\int_{0}^{t}\left(\alpha_{1}+\alpha_{2}+\mu \right)dt}dt\right].$$

Hence *S*(*t*) > 0 implying that S(t) is always positive, the same can be shown in a similar way that *D*(*t*) > 0, *M*(*t*) > 0, *Dm*(*t*) > 0, *R*_*d*_(*t*) > 0, *R*_*m*_(*t*) > 0, *R*_*dm*_(0) > 0, as t approaches ∞.

### 3.3. Drinkers model only

To get the model for drinkers only model, we set *R*_*m*_ = 0, *R*_*dm*_ = 0, *M* = 0, *Dm* = 0, *α*_2_ = 0, *γ*_2_ = 0, *γ*_3_ from Eq (1),

$$\begin{array}{c} \begin{array}{cc} & \frac{dS}{dt}=\xi +\gamma_{1}R_{d}-\left(\alpha_{1}+\mu \right)S,\\ & \frac{dD}{dt}=\alpha_{1}S-\left(\mu +\phi_{1}+\delta_{1}\right)D,\\ & \frac{dR_{d}}{dt}=\delta_{1}D-\left(\mu +\gamma_{1}\right)R_{d}. \end{array}\} \end{array}$$ (3)

#### 3.3.1. Drinking free equilibrium(DFE)

At DFE:

$$S\left(0\right)>0,\;D\left(0\right)=0,\;R_{d}=0.$$

$$∴DFE=\left(S^{*},D^{*},R_{d}^{*}\right)=(\frac{\xi }{\mu },0,0).$$

#### 3.3.2. Basic reproduction number of drinkers ($R_{0D}$)

The basic reproductive number is known to be the mean number of secondary influences that a single individual in the drinkers or smokers compartment has on the susceptible (vulnerable) population to drink or smoke or co-depend on both. Therefore, this research uses the next-generation matrix method to obtain the basic reproductive numbers.

For more people to be drinkers, the basic reproductive number for drinkers must be greater than one, that is, $R_{0D}>1$. Therefore, considering the drinkers class from Eq (3),

$$\frac{dD}{dt}=a_{1}DS-\left(\mu +\phi_{1}+\delta_{1}\right)D.$$

For more people to be drinkers, then $\frac{dD}{dt}>0,$ that is,

$$a_{1}DS-\left(\mu +\phi_{1}+\delta_{1}\right)D>0,$$

$$a_{1}DS>\left(\mu +\phi_{1}+\delta_{1}\right)D,$$

dividing both sides by (*μ* + *ϕ*_1_ + *δ*_1_)*D*, leads to,

$$\begin{array}{c} \begin{array}{c} \frac{a_{1}S}{\left(\mu +\phi_{1}+\delta_{1}\right)}>1. \end{array} \end{array}$$ (4)

By comparing Eq (4) to *R*_0*D*_ > 1,

$$R_{0D}=\frac{a_{1}S}{\left(\mu +\phi_{1}+\delta_{1}\right)},$$

and at drinking free equilibrium $S=\frac{\xi }{\mu }$. Hence the basic reproduction number of drinkers only is given as

$$R_{0D}=\frac{a_{1}\xi }{\mu (\mu +\phi_{1}+\delta_{1})}.$$

#### 3.3.3. Local stability of drinking free equilibrium(DFE)

**Theorem 3.2**. *The local asymptotic stability of the drinking free equilibrium point (DFE) is established when the basic reproduction number* ($R_{0}$) *for drinkers is below unity*, ($R_{0D}<1$) *and unstable when the value of* $R_{0D}$ *exceeds unity*.

*Proof*. By using the equations of the drinking model from Eq (3) and substituting *α*_1_ = *a*_1_*D*

$$\begin{array}{c} \begin{array}{cc} & \frac{dS}{dt}=\xi +\gamma_{1}R_{d}-\left(a_{1}D+\mu \right)S,\\ & \frac{dD}{dt}=a_{1}DS-\left(\mu +\phi_{1}+\delta_{1}\right)D,\\ & \frac{dR_{d}}{dt}=\delta_{1}D-\left(\mu +\gamma_{1}\right)R_{d}. \end{array}\} \end{array}$$ (5)

At drinking-free equilibrium, the Jacobian is obtained as

$$\begin{array}{c} J_{DFE}=[\begin{array}{ccc} -\mu & \frac{-a_{1}\xi }{\mu } & \gamma_{1}\\ 0 & \frac{a_{1}\xi }{\mu }-\left(\mu +\phi_{1}+\delta_{1}\right) & 0\\ 0 & 0 & -(\mu +\gamma_{1}) \end{array}]. \end{array}$$ (6)

The characteristic polynomial of Eq (6) is given as

$$\begin{array}{c} \left(-\mu -\lambda \right)((\frac{a_{1}\xi }{\mu }-\left(\mu +\phi_{1}+\delta_{1}\right))-\lambda )\left(-\left(\mu +\gamma_{1}\right)-\lambda \right)=0. \end{array}$$ (7)

The eigenvalues of Eq (7) is gotten as

$$\Rightarrow \lambda_{1}=-\mu ,\;\lambda_{2}=\frac{a_{1}\xi }{\mu }-\left(\mu +\phi_{1}+\delta_{1}\right),\;\lambda_{3}=-\left(\mu +\gamma_{1}\right),$$

hence for the system to be locally asymptotically stable at the drinking-free equilibrium then, λ_2_ must be less than zero, λ_2_ < 0. That is

$$\frac{a_{1}\xi }{\mu }-\left(\mu +\phi_{1}+\delta_{1}\right)<0,$$

hence,

$$\lambda_{2}=\frac{a_{1}\xi }{\mu }<\left(\mu +\phi_{1}+\delta_{1}\right),$$

$$\Rightarrow \lambda_{2}=\frac{a_{1}\xi }{\mu (\mu +\phi_{1}+\delta_{1})}=R_{0D}<1.$$

This implies that the DFE is asymptotically stable if $R_{0D}<1$, (basic reproductive number of drinking only is less than one), that is

$$R_{0D}=\frac{a_{1}\xi }{\mu (\mu +\phi_{1}+\delta_{1})}<1.$$

#### 3.3.4. Endemic equilibrium points for drinkers only model

Endemic Equilibrium occurs when drinking exists in the population, that is $R_{0D}>1$. Finding the endemic equilibrium points $\left(S^{*},D^{*},R_{D}^{*}\right)$, we set the drinkers model in Eq (3) to zero.

The endemic equilibrium points for drinkers is given as $\left(S^{*},D^{*},R_{d}^{*}\right)$

$$=(\frac{\left(\mu +\phi_{1}+\delta_{1}\right)}{a_{1}},\;\frac{\xi \left(\mu +\gamma_{1}\right)a_{1}-\left(\mu +\gamma_{1}\right)\left(\mu +\phi_{1}+\delta_{1}\right)\mu }{a_{1}\left(\left(\mu +\gamma_{1}\right)\left(\mu +\phi_{1}+\delta_{1}\right)-\gamma_{1}\delta_{1}\right)},\;\frac{\left(\mu +\gamma_{1}\right)a_{1}\delta_{1}\xi -\delta_{1}}{\left(\mu +\gamma_{1}\right)a_{1}\left(\left(\mu +\gamma_{1}\right)\left(\mu +\phi_{1}+\delta_{1}\right)-\gamma_{1}\delta_{1}\right)}).$$

#### 3.3.5. Global stability for the endemic equilibrium of drinkers model

**Theorem 3.3**. *The endemic equilibrium point (EEP) for drinkers only model is globally asymptotically stable if* $R_{0}$ *for drinkers is greater than one, that is* $R_{0D}>1$.

*Proof*. A Lyapunov function G according to [40] is defined such that

$$G=\left(S-S^{*}+S^{*}\;\text{ln}\;\frac{S^{*}}{S}\right)+\left(D-D^{*}+D^{*}\;\text{ln}\;\frac{D^{*}}{D}\right)+\left(R_{d}-R_{d}^{*}+R_{d}^{*}+\text{ln}\frac{R_{d}^{*}}{R_{d}}\right),$$

differentiating G with respect to t,

$$\begin{array}{c} \frac{dG}{dt}=(\frac{S-S^{*}}{S})\frac{dS}{dt}+(\frac{D-D^{*}}{D})\frac{dD}{dt}+(\frac{R_{d}-R_{d}^{*}}{R_{d}})\frac{dR_{d}}{dt}, \end{array}$$ (8)

and making substitution of $\frac{dS}{dt},\frac{dD}{dt},\frac{dR_{d}}{dt}$ from Eqs (3) into (8)

$$\begin{array}{c} \frac{dG}{dt}=(\frac{S-S^{*}}{S})\left(\xi +\gamma_{1}R_{d}-\left(\mu +\alpha_{1}\right)S\right)+(\frac{D-D^{*}}{D})\left(\delta_{1}S-\left(\mu +\phi_{1}+\delta_{1}\right)D\right)+\\ (\frac{R_{d}-R_{d}^{*}}{R_{d}})\left(\delta_{1}D-\left(\mu +\gamma_{1}\right)R_{d}\right). \end{array}$$ (9)

By expanding and grouping terms,

$$\begin{array}{ccc} \frac{dG}{dt} & = & \left(\xi +\gamma_{1}R_{d}+\alpha_{1}S^{*}+\mu S^{*}+\alpha_{1}S+\mu D^{*}+\phi D^{*}+\delta_{1}D^{*}+\delta_{1}D+\mu R_{d}^{*}+\gamma_{1}R_{d}^{*}\right)\\ & & -(\alpha_{1}S+\mu S+\frac{\xi S^{*}}{S}+\frac{\gamma_{1}R_{d}S^{*}}{S}+\mu D+\phi_{1}D+\delta_{1}D+\frac{\alpha_{1}SD^{*}}{D}+\mu R_{d}+\gamma_{1}R_{d}+\frac{\delta_{1}DR_{d}^{*}}{R_{d}}). \end{array}$$

$\Rightarrow \frac{dG}{dt}=G_{1}-G_{2}$, where,

$$\begin{array}{c} G_{1}=(\xi +\gamma_{1}R_{d}+\alpha_{1}S^{*}+\mu S^{*}+\alpha_{1}S+\mu D^{*}+\phi_{1}D^{*}+\delta_{1}D^{*}+\delta_{1}D+\mu R_{d}^{*}+\gamma_{1}R_{d}^{*}), \end{array}$$

$$\begin{array}{c} G_{2}=(\alpha_{1}S+\mu S+\frac{\xi S^{*}}{S}+\frac{\gamma_{1}R_{d}S^{*}}{S}+\mu D+\phi_{1}D+\delta_{1}D+\frac{\alpha_{1}SD^{*}}{D}+\mu R_{d}+\gamma_{1}R_{d}+\frac{\delta_{1}DR_{d}^{*}}{R_{d}}). \end{array}$$

According to [40] If *G*_1_ < *G*_2_ then $\frac{dG}{dt}$ will be negative which means the endemic equilibrium is asymptotically stable and unstable otherwise.

### 3.4. Smokers model only

To get the model of smokers only we set *D* = 0, *R*_*d*_ = 0, *Dm* = 0, *R*_*dm*_ = 0, *α*_1_ = 0, *γ*_1_ = 0 from Eq (1) to obtained

$$\begin{array}{c} \begin{array}{cc} & \frac{dS}{dt}=\xi +\gamma_{2}R_{m}-{\left(\mu +\alpha_{2}\right)}_{S},\\ & \frac{dM}{dt}=\alpha_{2}S-\left(\mu +\phi_{2}+\delta_{2}\right)M,\\ & \frac{dR_{m}}{dt}=\delta_{2}M-\left(\mu +\gamma_{2}\right)R_{m}. \end{array}\} \end{array}$$ (10)

#### 3.4.1. Smoking free equilibrium points(MFE)

At smoking free equilibrium *S*(0) ≥ 0, *M*(0) = 0, *R*_*m*_(0) = 0. Therefore the MFE is

$$\left(S^{*},M^{*},R_{m}^{*}\right)=\left(\frac{\xi }{\mu },0,0\right).$$

#### 3.4.2. Basic reproductive number for smoking ($R_{0m}$)

Smoking exists in the population if the basic reproduction number of smoking is greater than one, that is $R_{0M}<1$. From Eq (10). Considering the smoking class

$$\frac{dM}{dt}=a_{2}MS-\left(\mu +\phi_{2}+\delta_{2}\right)M.$$

For more people to engage in smoking, $\frac{dM}{dt}>0,$ hence

$$a_{2}MS>\left(\mu +\phi_{2}+\delta_{2}\right)M\;\text{implying}\;\text{that}$$

$$\begin{array}{c} \begin{array}{c} \frac{a_{2}S}{\left(\mu +\phi_{2}+\delta_{2}\right)}>1. \end{array} \end{array}$$ (11)

By comparing Eq (11) to $R_{0}>1$ then,

$$R_{0M}=\frac{a_{2}S}{\left(\mu +\phi_{2}+\delta_{2}\right)},$$

but at smoking free equilibrium $S=\frac{\xi }{\mu },$ hence

$$R_{0M}=\frac{\xi a_{2}}{\mu (\mu +\phi_{2}+\delta_{2})}.$$

#### 3.4.3. Local stability of smoking-free equilibrium

**Theorem 3.4**. *The smoking-free equilibrium point for smoking is locally asymptotically stable if* $R_{0M}<1$ *and unstable if* $R_{0M}>1$

*Proof*. From Eq (10). The Jacobian of the smoking-free equilibrium is

$${\text{J}}_{\text{MFE}}=[\begin{array}{ccc} -\mu & -a_{2}\frac{\xi }{\mu } & \gamma_{2}\\ a_{2}M & a_{2}\frac{\xi }{\mu }-\left(\mu +\phi_{2}+\delta_{2}\right) & 0\\ 0 & \delta_{2} & \left(\mu +\gamma_{2}\right) \end{array}],$$

and the characteristic polynomial is given by

$$\left(-\mu -\lambda \right)\;((a_{2}\frac{\xi }{\mu }-\left(\mu +\phi_{2}+\delta_{2}\right))-\lambda )\left(-\left(\mu +\gamma_{2}\right)-\lambda \right)=\;0,$$

with eigenvalues gotten as

$$\lambda_{1}=-\mu ,\;\lambda_{2}=x_{2}=-\left(\mu +\gamma_{2}\right),\;\lambda_{3}=\frac{a_{2}\xi }{\mu }-\left(\mu +\phi_{2}+\delta_{2}\right).$$

For the smoking-free equilibrium to be locally asymptotically stable λ_3_ < 0, thus

$$\frac{a_{2}\xi }{\mu }-\left(\mu +\phi_{2}+\delta_{2}\right)<0,$$

which implies that

$$\frac{a_{2}\xi }{\mu }<\left(\mu +\phi_{2}+\delta_{2}\right).$$

Dividing through by (*μ* + *ϕ*_2_ + *δ*_2_),

$$\frac{a_{2}}{\mu (\mu +\phi_{2}+\delta_{2})}=R_{0M}<1.$$

Therefore the smoking-free equilibrium is locally asymptotically stable if $R_{0M}<1$, that is the basic reproductive number for smoking is less than 1.

#### 3.4.4. Endemic equilibrium points for smoking model

Endemic Equilibrium occurs when smoking exists in the population, that is $R_{0M}>1$. Finding the endemic equilibrium points $(S^{*},M^{*},R_{m}^{*}).$ We set the smoking model Eq (10) to zero. Hence we obtained the endemic equilibrium points for smoking as $\left(S^{*},M^{*},R_{d}^{*}\right)$

$$=(\frac{\left(\mu +\phi_{2}+\delta_{2}\right)}{a_{2}},\;\frac{\xi \left(\mu +\gamma_{2}\right)a_{2}-\left(\mu +\gamma_{2}\right)\left(\mu +\phi_{2}+\delta_{2}\right)\mu }{a_{2}\left(\left(\mu +\gamma_{2}\right)\left(\mu +\phi_{2}+\delta_{2}\right)-\gamma_{2}\delta_{2}\right)},\;\frac{\left(\mu +\gamma_{2}\right)a_{2}\delta_{2}\xi -\delta_{2}}{\left(\mu +\gamma_{2}\right)a_{2}\left(\left(\mu +\gamma_{2}\right)\left(\mu +\phi_{2}+\delta_{2}\right)-\gamma_{2}\delta_{2}\right)}).$$

#### 3.4.5. Global stability for the endemic equilibrium of smoking model

**Theorem 3.5**. *The endemic equilibrium points(EEP) for smoking is asymptotically stable if* $R_{0M}>1.$

We define a Lyapunov function G, according to [40] such that

$$G=(S-S^{*}+S^{*}\;\text{ln}\;\frac{S^{*}}{S})+(D-D^{*}+D^{*}\;\text{ln}\;\frac{D^{*}}{D})+(R_{d}-R_{d}^{*}+R_{d}^{*}+\;\text{ln}\;\frac{R_{d}^{*}}{R_{d}}),$$

differentiating the function G with respect to t

$$\begin{array}{c} \frac{dG}{dt}=(\frac{S-S^{*}}{S})\frac{dS}{dt}+(\frac{M-M^{*}}{M})\frac{dM}{dt}+(\frac{R_{m}-R_{m}^{*}}{R_{m}})\frac{dR_{m}}{dt}, \end{array}$$ (12)

making substitution of $\frac{dS}{dt},\frac{dM}{dt},\frac{dR_{m}}{dt},$ from Eq (10) into Eq (12)

$$\begin{array}{c} \frac{dG}{dt}=(\frac{S-S^{*}}{S})\left(\xi +\gamma_{2}R_{m}-\left(\mu +\alpha_{2}\right)S\right)+(\frac{M-M^{*}}{M})\left(\delta_{2}S-\left(\mu +\phi_{2}+\delta_{2}\right)M\right)+(\frac{R_{m}-R_{m}^{*}}{R_{m}})\left(\delta_{2}M-\left(\mu +\gamma_{2}\right)R_{m}\right). \end{array}$$ (13)

By expanding and grouping terms

$$\frac{dG}{dt}=(\xi +\gamma_{2}R_{m}+\alpha_{2}S^{*}+\mu S^{*}+\mu m^{*}+\phi_{2}M^{*}+\delta_{2}M^{*}+\delta_{2}M+\frac{\delta_{2}MR_{m}^{*}}{R_{m}}+\mu R_{m}^{*}+\gamma_{2}R_{m}^{*})-$$

$$(\alpha_{2}S+\mu S+\frac{\xi S^{*}}{S}+\frac{\gamma_{2}R_{m}S^{*}}{S}+\mu M+\phi_{2}M+\delta_{2}M+\frac{\alpha_{2}SM^{*}}{M}+\mu R_{m}+\gamma_{2}R_{m}),$$

$\Rightarrow \frac{dG}{dt}=G_{1}-G_{2},$ where

$$G_{1}=(\xi +\gamma_{2}R_{m}+\alpha_{2}S^{*}+\mu S^{*}+\mu M^{*}+\phi_{2}M^{*}+\delta_{2}M^{*}+\delta_{2}M+\frac{\delta_{2}MR_{m}^{*}}{R_{m}}+\mu R_{m}^{*}+\gamma_{2}R_{m}^{*}),$$

$$G_{2}=(\alpha_{2}S+\mu S+\frac{\xi S^{*}}{S}+\frac{\gamma_{2}R_{m}S^{*}}{S}+\mu M+\phi_{2}M+\delta_{2}M+\frac{\alpha_{2}SM^{*}}{M}+\mu R_{m}+\gamma_{2}R_{m}).$$

If *G*_1_ < *G*_2_ then $\frac{dG}{dt}$ will be negative which means that the endemic equilibrium points is asymptotically stable and unstable otherwise.

### 3.5. Drinking and smoking co-dependence model

The following qualitative analysis is performed on the co-dependence of drinking and smoking using Eq (1).

#### 3.5.1. Drinking and smoking free equilibrium(DMFE)

At the drinking and smoking free equilibrium, we set *D* = 0, *M* = 0, *Dm* = 0, *R*_*d*_ = 0, *R*_*m*_ = 0, *R*_*dm*_ = 0 from Eq (1). From the first equation, we set $\frac{dS}{dt}=0,then$

$$\begin{array}{c} \frac{dS}{dt}=\xi +\gamma_{1}R_{d}+\gamma_{2}R_{m}+\gamma_{3}R_{dm}-\left(\alpha_{1}+\alpha_{2}+\mu \right)S, \end{array}$$ (14)

substituting *D* = 0, *M* = 0, *Dm* = 0, *R*_*d*_ = 0, *R*_*m*_ = 0, *R*_*dm*_ = 0 into Eq (14)

0=ξ-μS,

$$\Rightarrow S\left(t\right)=\frac{\xi }{\mu },$$

$$∴DSFE=\left(S^{*},D^{*},M^{*},{Dm}^{*},R_{d}^{*},R_{m}^{*},R_{dm}^{*}\right)=(\frac{\xi }{\mu },0,0,0,0,0,0).$$

#### 3.5.2. Basic reproductive number of the co-dependence on drinking and smoking

By considering the infective compartment of the model in Eq (1)

$$\begin{array}{ccc} \frac{dD}{dt} & = & a_{1}DS+a_{1}DmS-\left(\mu +\phi_{1}+\delta_{1}+\alpha_{2}\right)D,\\ \frac{dM}{dt} & = & a_{2}MS+a_{2}DmS-\left(\mu +\phi_{2}+\delta_{2}+\alpha_{1}\right)M,\\ \frac{dDm}{dt} & = & a_{2}MD+a_{2}DmD+a_{1}DM+a_{1}DmM-\left(\mu +\phi_{3}+k_{1}\right)Dm. \end{array}$$

We then use the next generation matrix with the definitions F and V;

$$\begin{array}{c} F=(\begin{array}{c} a_{1}DS+a_{1}DmS\\ a_{2}MS+a_{2}DmtS\\ 0 \end{array})\;\text{and}\;V=(\begin{array}{c} (\mu +\phi_{1}+\delta_{1}+\alpha_{2})D\\ (\mu +\phi_{2}+\delta_{2}+\alpha_{1})M\\ -\left(a_{2}MD+a_{2}DmD+a_{1}DM+a_{1}DmM\right)+\left(\mu +\phi_{3}+k_{1}\right)Dm \end{array}), \end{array}$$

The matrices above are then linearized by using the Jacobian matrix to obtain *F* and *V* as;

$$\begin{array}{c} F=(\begin{array}{ccc} a_{1}S & 0 & a_{1}S\\ 0 & A_{2}S & a_{2}S\\ 0 & 0 & 0 \end{array})\;\text{and}\;V=(\begin{array}{ccc} \left(\mu +\phi_{1}+\delta_{1}\right) & 0 & 0\\ 0 & \left(\mu +\phi_{2}+\delta_{2}\right) & 0\\ 0 & 0 & (\mu +\phi_{3}+k_{1} \end{array}), \end{array}$$

and through mathematical computations, we have;

$$FV^{-1}=[\begin{array}{ccc} \frac{a_{1}\xi }{\mu (\mu +\phi_{1}+\delta_{1})} & 0 & \frac{a_{1}\xi }{\mu (\mu +\Phi_{3}+k_{1})}\\ 0 & \frac{a_{2}\xi }{\mu (\mu +\phi_{2}+\delta_{2})} & \frac{a_{2}\xi }{\mu (\mu +\phi_{3}+k_{1})}\\ 0 & 0 & 0 \end{array}].$$

The eigenvalues of the matrix above are computed through the concept of the determinant of a matrix. This yields;

$$\begin{array}{c} det\left(FV^{-1}\right)=|FV^{1}-\lambda I|=|\begin{array}{ccc} \frac{a_{1}\xi }{\mu (\mu +\phi_{1}+\delta_{1})}-\lambda & 0 & \frac{a_{1}\xi }{\mu (\mu +\Phi_{3}+k_{1})}\\ 0 & \frac{a_{2}\xi }{\mu (\mu +\phi_{2}+\delta_{2})}-\lambda & \frac{a_{2}\xi }{\mu (\mu +\phi_{3}+k_{1})}\\ 0 & 0 & -\lambda \end{array}|. \end{array}$$ (15)

Hence the eigenvalues of Eq (15) are

$$\lambda_{1}=\frac{a_{1}\xi }{\mu (\mu +\phi_{1}+\delta_{1})},\;\lambda_{2}=\frac{a_{2}\xi }{\mu (\mu +\phi_{2}+\delta_{2})},\lambda_{3}=0,$$

and the dominant eigenvalue which is referred to as the basic reproductive number, *ρ*(*FV*^−1^) is given as;

$$\lambda_{1}=\frac{a_{1}\xi }{\mu (\mu +\phi_{1}+\delta_{1})},\;\lambda_{2}=\frac{a_{2}\xi }{\mu (\mu +\phi_{2}+\delta_{2})}.$$

Therefore the basic reproductive number is given by $R_{0}=\text{sup}\left\{\lambda_{1},\lambda_{2}\right\}$.

#### 3.5.3 Local stability drinking and smoking free equilibrium

**Theorem 3.6**. *The stability at the drinking and smoking-free equilibrium is locally asymptotically stable if* $R_{0}\leq 0$ *and unstable if* $R_{0}\geq 1$.

*Proof*. At DMFE, the Jacobian matrix is obtained as

$$\begin{array}{c} J_{DMFE}=[\begin{array}{ccccccc} -\mu & \frac{-a_{1}\xi }{\mu } & \frac{-a_{2}\xi }{\mu } & 0 & \gamma_{1} & \gamma_{2} & \gamma_{3}\\ 0 & \frac{-a_{1}\xi }{\mu }-\left(\mu +\phi_{1}+\delta_{1}\right) & 0 & \frac{-a_{1}\xi }{\mu } & 0 & 0 & 0\\ 0 & 0 & \frac{-a_{1}\xi }{\mu }-\left(\mu +\phi_{2}+\delta_{2}\right) & \frac{-a_{2}\xi }{\mu } & 0 & 0 & 0\\ 0 & 0 & 0 & -(\mu +\phi_{3}+k_{1}) & 0 & 0 & 0\\ 0 & \delta_{1} & 0 & 0 & -(\mu +\gamma_{1}) & 0 & 0\\ 0 & 0 & \delta_{2} & 0 & 0 & -(\mu +\gamma_{2}) & 0\\ 0 & 0 & 0 & k_{1} & 0 & 0 & -(\mu +\gamma_{3}) \end{array}]. \end{array}$$ (16)

From Eq (16) the characteristic polynomial is obtained as

$$\begin{array}{ccc} & & \left(-\mu -\lambda \right)\left(\left(\frac{-a_{1}\xi }{\mu }-\left(\mu +\phi_{1}+\delta_{1}\right)\right)-\lambda \right)\left(\left(\frac{-a_{1}\xi }{\mu }-\left(\mu +\phi_{2}+\delta_{2}\right)\right)-\lambda \right)\left(-\left(\mu +\phi_{3}+k_{1}\right)-\lambda \right),\\ & & \left(-\left(\mu +\gamma_{1}\right)-\lambda \right)\left(-\left(\mu +\gamma_{2}\right)-\lambda \right)\left(-\left(\mu +\gamma_{1}\right)-\lambda \right)=0. \end{array}$$

Hence

$$\begin{array}{ccc} & & \lambda_{0}=-\mu ,\;\lambda_{1}=\frac{a_{1}\xi }{\mu }-\left(\mu +\phi_{1}+\delta_{1}\right),\;\lambda_{2}=\frac{a_{1}\xi }{\mu }-\left(\mu +\phi_{2}+\delta_{2}\right),\;\lambda_{3}=-\left(\mu +\phi_{3}+k_{1}\right),\\ & & \lambda_{4}=-\left(\mu +\gamma_{1}\right),\;\lambda_{5}=-\left(\mu +\gamma_{2}\right)\;\lambda_{6}=-\left(\mu +\gamma_{2}\right). \end{array}$$

This implies that the drinking and smoking-free equilibrium can only be stable if λ_1_ and λ_2_ are negative, that is, λ_1_ < 0 and λ_2_ < 0. Therefore, from $\frac{-a_{1}\xi }{\mu }+\left(\mu +\phi_{1}+\delta_{1}\right)=\lambda_{1},$ if λ_1_ < 0 then

$$\begin{array}{c} \begin{array}{c} \frac{a_{1}\xi }{\mu }<\left(\mu +\phi_{1}+\delta_{1}\right), \end{array} \end{array}$$ (17)

dividing Eq (17) by (*μ* + *ϕ*_1_ + *δ*_1_), we obtained;

$$\frac{a_{1}\xi }{\mu (\mu +\phi_{1}+\delta_{1})}<1,$$

which is $R_{0}$ for drinkers, that is,

$$R_{0D}=\frac{a_{1}\xi }{\mu (\mu +\phi_{1}+\delta_{1})}.$$

Also if λ_2_ < 0, from $\frac{a_{2}\xi }{\mu }-\left(\mu +\phi_{2}+\delta_{2}\right)=\lambda_{2}$, then we have,

$$\begin{array}{c} \begin{array}{c} \frac{a_{2}\xi }{\mu }<\left(\mu +\phi_{2}+\delta_{2}\right), \end{array} \end{array}$$ (18)

dividing Eq (18) by (*μ* + *ϕ*_2_ + *δ*_2_), we obtained

$$\frac{a_{2}\xi }{\mu (\mu +\phi_{2}+\delta_{2})}<1,$$

which is $R_{0}$ for Smokers, that is,

$$R_{0M}=\frac{a_{2}\xi }{\mu (\mu +\phi_{2}+\delta_{2})}.$$

Hence the drinking and smoking-free equilibrium will be asymptotically stable if $R_{0D}<1$ and $R_{0M}<1$.

#### 3.5.4. Global stability of drinking and smoking-free equilibrium points(DMFE)

To ascertain the global stability of the equilibrium state characterized by abstaining from drinking and smoking, we employ the computational approach proposed by Castillo-Chavez [41]. The drinking and smoking model is’ written as

$$\frac{dX}{dt}=F\left(X,Z\right),$$

$$\frac{dZ}{dt}=G\left(X,Z\right),\;G\left(X,0\right),$$

where X is the non-drinkers and smokers population, that is, *X* = (*S*, *R*_*d*_, *R*_*m*_, *R*_*dm*_), and *Z* is the drinkers and smokers population, thus *Z* = (*D*, *M*, *Dm*). The equilibrium point where drinking and smoking are both absent is represented by the notation *Q* = (X*, 0). Point Q exhibits global asymptotic stability under the condition that *R*_0_ is less than 1, and both step I and step II are satisfied.

step I. $\frac{dX}{dt}=F\left(X,0\right),\;X^{*}$ is globally asymptotically stable.

step II. $G\left(X,Z\right)=AZ-\tilde{G}\left(X,Z\right),\tilde{\left(X,Z\right)}\geq 0\;\text{for}\left(X,Z\right)$.

**Theorem 3.7**. *The equilibrium point Q* = (X*, 0) *can be considered to be global asymptotically stable if the value of* $R_{0}$ *is less than 1 and if criteria (I) and (II) are met*.

From Eq (1) the non-drinkers and smokers class is given as

$$F\left(X,Z\right)=(\begin{array}{c} \xi +\gamma_{1}R_{d}+\gamma_{2}R_{m}+\gamma_{3}R_{dm}-\left(\alpha_{1}+\alpha_{2}+\mu \right)S\\ \delta_{1}D-\left(\mu +\gamma_{1}\right)R_{d}\\ \delta_{2}M-\left(\mu +\gamma_{2}\right)R_{m}\\ K_{1}Dm-\left(\mu +\gamma_{3}\right)R_{dm} \end{array}),$$

at the DMFE

$$F\left(X,0\right)=(\begin{array}{c} \xi -\mu S\\ 0\\ 0\\ 0 \end{array}).$$

and the drinkers and smokers class is obtained as

$$G\left(X,Z\right)=(\begin{array}{c} \alpha_{1}S-\left(\mu +\phi_{1}+\delta_{1}+\alpha_{2}\right)D\\ \alpha_{2}S-\left(\mu +\phi_{2}+\delta_{2}+\alpha_{1}\right)M\\ \alpha_{2}D+\alpha_{1}M-\left(\mu +\phi_{3}+k_{1}\right)Dm \end{array}).$$

We linearized *G*(*X*, *Z*) using the Jacobian and acquired *J*_*G*(*X*,*Z*)_ as

$$J_{G(X,Z)}=A=(\begin{array}{ccc} -(\mu +\phi_{1}+\delta_{1}+\alpha_{2}) & 0 & 0\\ 0 & -(\mu +\phi_{2}+\delta_{2}+\alpha_{1}) & 0\\ \alpha_{2} & \alpha_{1} & -(\mu +\phi_{3}+k_{1}) \end{array}),$$

but from step II, $\tilde{G}\left(X,Z\right)=AZ-G\left(X,Z\right),$ hence

$$\begin{array}{cc} \tilde{G}\left(X,Z\right) & =(\begin{array}{ccc} -(\mu +\phi_{1}+\delta_{1}+\alpha_{2}) & 0 & 0\\ 0 & -(\mu +\phi_{2}+\delta_{2}+\alpha_{1}) & 0\\ \alpha_{2} & \alpha_{1} & -(\mu +\phi_{3}+k_{1}) \end{array})(\begin{array}{c} D\\ M\\ Dm \end{array})\\ & -(\begin{array}{c} \alpha_{1}S-\left(\mu +\phi_{1}+\delta_{1}+\alpha_{2}\right)D\\ \alpha_{2}S-\left(\mu +\phi_{2}+\delta_{2}+\alpha_{1}\right)M\\ \alpha_{2}D+\alpha_{1}M-\left(\mu +\phi_{3}+k_{1}\right)Dm \end{array}), \end{array}$$

$$\begin{array}{c} \begin{array}{ccc} \tilde{G}\left(X,Z\right) & =\left(\begin{array}{c} -(\mu +\phi_{1}+\delta_{1}+\alpha_{2})D\\ -(\mu +\phi_{2}+\delta_{2}+\alpha_{1})M\\ \alpha_{2}D+\alpha_{1}M-\left(\mu +\phi_{3}+k_{1}\right)\left(Dm\right) \end{array})-(\begin{array}{c} \alpha_{1}S-\left(\mu +\phi_{1}+\delta_{1}+\alpha_{2}\right)D\\ \alpha_{2}S-\left(\mu +\phi_{2}+\delta_{2}+\alpha_{1}\right)M\\ \alpha_{2}D+\alpha_{1}M-\left(\mu +\phi_{3}+k_{1}\right)Dm \end{array}\right), & \\ \tilde{G}\left(X,Z\right) & =(\begin{array}{c} \alpha_{1}S\\ \alpha_{2}S\\ 0 \end{array}). & \end{array} \end{array}$$

Since condition (2) of Castillo Chavez is satisfied, that is, $\tilde{G}\geq 0$, then the drinking and smoking free is globally asymptotically stable if *R*_0_ < 1. This implies that the drinking and smoking model can be put under control no matter the number of individuals that initially drink and smoke; that is, drinking and smoking can gradually be eradicated.

## 4. Sensitivity index analysis

In this section, sensitivity analysis was conducted to determine the influential parameters in the model, specifically examining the robustness of parameter values on the basic reproduction number ($R_{0}$) by using numerical values in Table 2. The parameter values are obtained from the literature. The rest of the parameters are obtained as follows: From [42], it is indicated that approximately one-third of the individuals treated at the rehabilitation centres to quit alcohol intake recover by twelve months, that, within one to twelve months, the individuals no longer exhibit signs of drinking usage. Also, from [43], it is indicated that individuals in treatment centers for smoking improve their lung functioning, and this reduces the chance of smoking within twelve months. Therefore, we estimated *k*_1_ as $\frac{1}{52\;\;weeks}$. From the work of [44], *ϕ*_1_ is given as 0.000009 *weeks*^−1^ and Zaman et al. [45] gives *ϕ*_2_ = 0.175 *weeks*^−1^. Hence, we estimated *ϕ*_3_ = *ϕ*_1_ + *ϕ*_2_ as done in [35]. Furthermore, from the work of Agrawal et al. [46] it is indicated that, *γ*_1_ = 0.7 *weeks*^−1^ and *γ*_2_ is given as 0.00575 *weeks*^−1^ in [47], so it is estimated that $\gamma_{3}=\frac{\gamma_{1}+\gamma_{2}}{2}$. To study the sensitivity of the parameter in the basic reproduction number. We employ the forward sensitivity index, 3D plots, scatter plots, and Latin hypercube sampling. The forward sensitivity index of a parameter *x* with respect to $R_{0}$ is defined as $W_{x}^{R_{0}}=\frac{\partial R_{0}}{\partial x}\frac{x}{R_{0}}$, where *x* is the various parameters in the basic reproduction number. The respective sensitivity indexes using Table 2 are given in Table 3.

**Table 2:** Parameter values of the model.

Parameter symbol	Value	Source
*ξ*	0.8 *weeks*^−1^	Assumed
*γ*_1_	0.7 *weeks*^−1^	[46]
*a*_1_	0.00479 *weeks*^−1^	[48]
*μ*	0.0952 *weeks*^−1^	[45]
*ϕ*_1_	0.000009 *weeks*^−1^	[44]
*δ*_1_	0.00767 *weeks*^−1^	[44]
*γ*_2_	0.00575 *weeks*^−1^	[47]
*γ*_3_	0.352875 *weeks*^−1^	Estimated
*a*_2_	0.035 *weeks*^−1^	[49]
*ϕ*_2_	0.175 *weeks*^−1^	[45]
*ϕ*_3_	*ϕ*_1_ + *ϕ*_2_ *weeks*^−1^	Estimated
*δ*_2_	0.217 *weeks*^−1^	[45]
*k*_1_	0.01923 *weeks*^−1^	Estimated

**Table 3:** Sensitivity indices of parameter values.

Parameters	Sensitivity Index	Parameters	Sensitivity Index
*ξ*	+1		
$R_{0}$ for Drinking		$R_{0}$ for Smoking	
*a*_1_	+1	*δ*_2_	-0.075
*ϕ*_1_	-0.000087	*a*_2_	+1
*μ*	-1.925	*μ*	-1.1954
*δ*_1_	-0.4454	*ϕ*_2_	-0.3592

**Fig 2:**
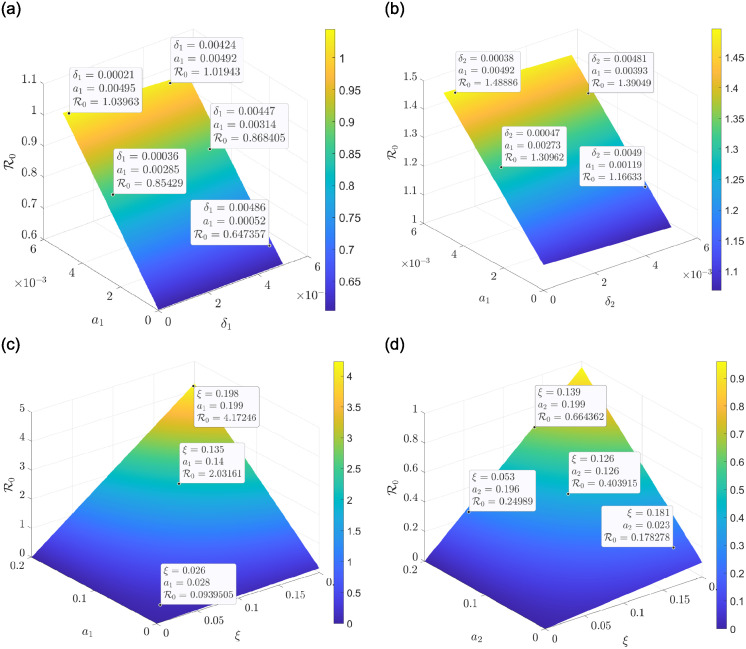
A three dimension sensitivity plots between $R_{0}$ and some parameters. (a) A surface plot on the behavior of $R_{0}$ under *a*_1_ and *δ*_1_. (b) A surface plot on the behavior of $R_{0}$ under *a*_1_ and *δ*_2_. (c) A surface plot on the behavior of $R_{0}$ under *a*_1_ and *ξ*. (d) A surface plot on the behavior of $R_{0}$ under *a*_2_ and *ξ*.

From Table 3, the parameters with a negative index have an impact on controlling the spread of drinking and smoking as they are inversely proportional to their respective $R_{0}$. Hence, increasing (*δ*_1_, *δ*_2_, *k*_1_, *μ*, *ϕ*_1_, *ϕ*_2_) has a positive influence in reducing $R_{0}$, which will lead to a decrease in the number of drinkers and smokers in the population. Fig 2a illustrates a downward behaviour in $R_{0}$ whenever we simultaneously reduce the contact rate (*a*_1_) and increase the recovery rate (*δ*_1_) of the drinkers’ population. This, therefore, indicates a decrease in the drinkers’ population. Also, Fig 2b shows that reducing the drinkers’ contact rate *a*_1_ and increasing the smokers’ recovery rate *δ*_2_ decreases the $R_{0}$ leading to a decrease in the co-dependence population. The results from Fig 2c indicate further that increasing the recruitment rate and the contact rate of drinkers leads to an increase in $R_{0}$, which will result in more people consuming alcohol. A similar analysis is observed for an increase in the contact rate of smokers and the recruitment rate when viewed from the same trajectory as in Fig 2d.

**Fig 3:**
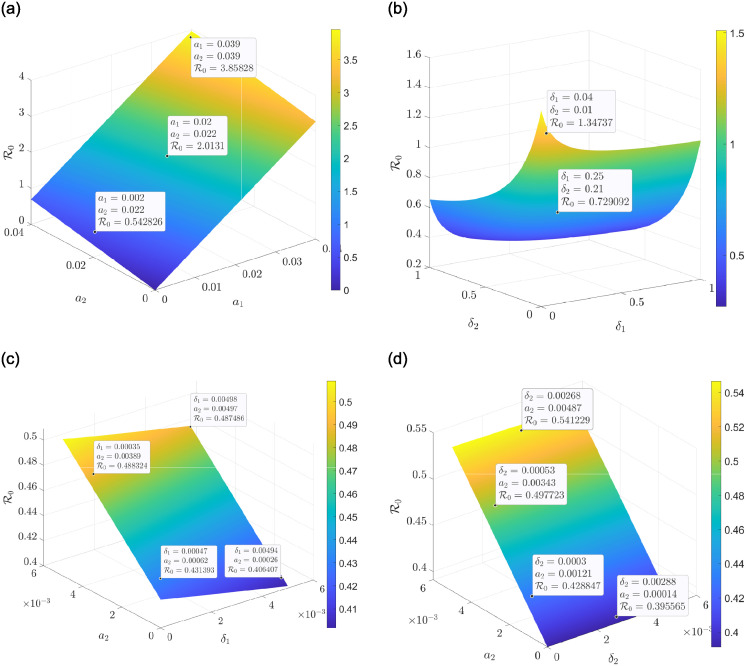
A three dimension sensitivity plots between $R_{0}$ and some parameters. (a) A surface plot on the behavior of $R_{0}$ under *a*_1_ and *a*_2_. (b) A surface plot on the behavior of $R_{0}$ under *δ*_2_ and *δ*_1_. (c) A surface plot on the behavior of $R_{0}$ under *a*_2_ and *δ*_1_. (d) A surface plot on the behavior of $R_{0}$ under *a*_2_ and *δ*_2_.

The 3D plots in Fig 3a and 3b also indicate that increasing both the contact rate of drinkers and smokers subsequently leads to an increase in the $R_{0}$ and increasing both the recovery rate of drinkers and smokers results in a decrease in the $R_{0}$. This, therefore, leads to a reduction in the co-dependence of alcohol and smoking consumption. Fig 3c clearly shows that as the contact rate of smokers increases and the recovery rate of drinkers is minimised, it will lead to a rise in the $R_{0}$, indicating an increase in the co-dependence of drinking and smoking. Similar results are obtained with the contact rate of smokers and the recovery rate of smokers, as shown in Fig 3d.

Scatter plots are employed to enable us to study how the model parameters relate to the basic reproductive number since they are posited in [50] to be a robust approach to establishing the exact relationship between objects. According to the concept of correlation, any parameter that yields a correlation pattern in a scatter plot indicates a relationship. Also, the Monte Carlo technique, the Latin Hypercube Sampling (LHS) technique, is employed to quantify the parameter value uncertainties and measure how sensitive the parameters may be in the model. Thus, LHS will help us establish the degree to which the parameters exert influence on the spread of drinking and smoking infections and their co-dependence. In addition, to help measure the non-linear but monotonous relationship among the model parameters, the LHS is applied hand in hand with a Partial Rank Correlation Coefficient (PRCC) [51]. Through the PRCC, tornado plots are performed to study how the parameters in the model are correlated to the state variables *D*, *M*, and *Dm*. Parameters that are more significant have PRCC values of either (0.5, 1) or (0, −1), which further indicates a strong positive or negative correlation. For detailed scatter plots and PRCC notes, refer to [50, 52].

**Fig 4:**
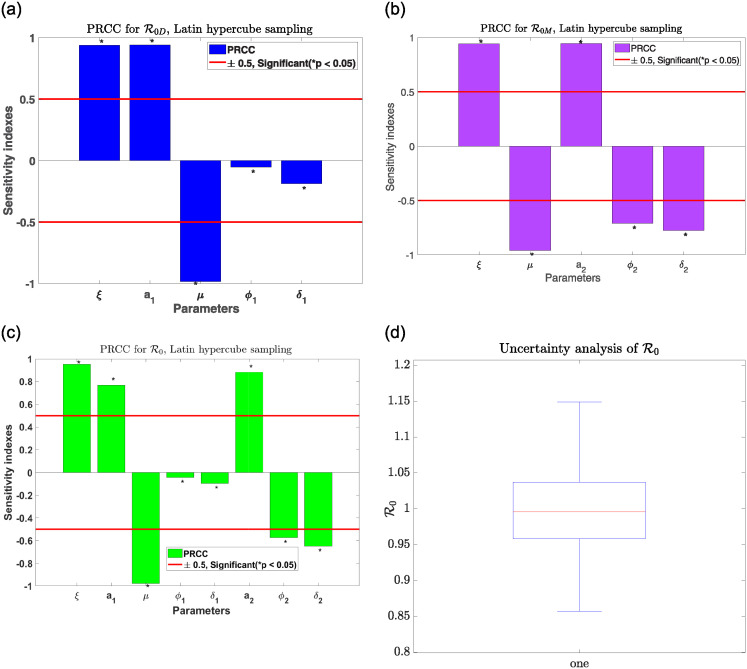
Global sensitivity and $R_{0}$ unpredictability analysis. (a) partial rank correlation plot for drinking only. (b) partial rank correlation plot for smoking only. (c) partial rank correlation plot for drinking and smoking co-dependence. (d) $R_{0}$ box graph for drinking and smoking co-dependence.

In Fig 4, the sensitivity and unpredictability behaviour of the obtained basic reproductive number for drinkers only, smokers only, and drinkers and smokers co-dependence are extensively shown using the Monte Carlo PRCC approach. Thus, the unpredictable nature of the reproduction number is studied, and the degree of uncertainty is measured through a 95% confidence interval. It is shown in Fig 4a that the parameters *ξ* and *a*_1_ contribute to the spread of individuals drinking, whereas, in a significant manner, *μ* helps in the reduction in the number of individuals depending on smoking. It is also evident in Fig 4b that *ξ* and *a*_2_ account for an increase in the number of individuals that consume alcoholic drinks while on the other hand, *μ* followed by *δ*_2_ and *ϕ*_1_ helps in the reduction of alcohol drink consumption. The co-dependence on both drinking and smoking is observed to increase significantly at the introduction of the parameters *ξ*, *a*_1_ and *a*_2_. In contrast, the co-dependence is reduced significantly for the given parameters *μ*, *δ*_2_ and *ϕ*_2_ as seen in Fig 4c. From Fig 4d, it is explicitly shown that the uncertainty analysis seen observed in $R_{0}$ lies in the interval ([0.8−1.2]) as many of the parameters are found in the high ranges.

**Fig 5:**
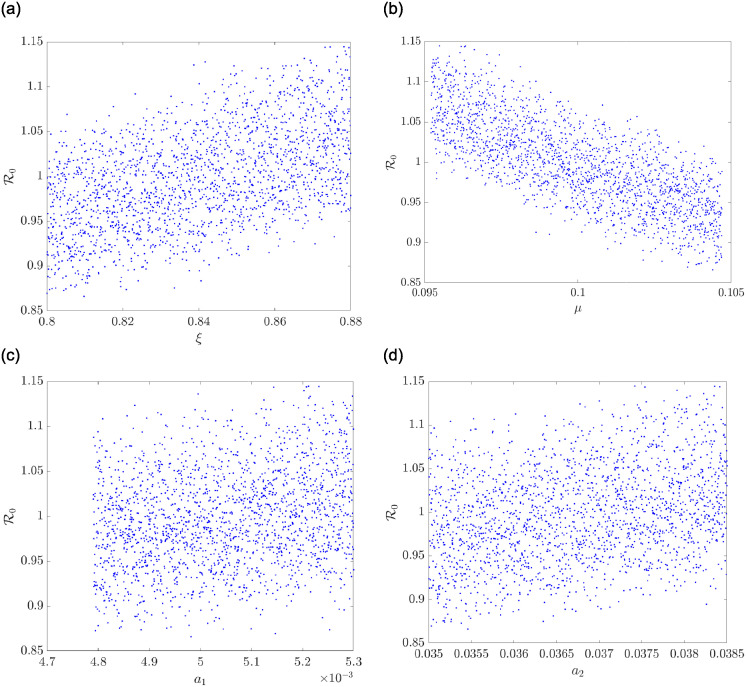
A scatter correlation plot for some parameters. (a) A scatter correlation plot for *ξ* and $R_{0}$. (b) A scatter correlation plot for *μ* and $R_{0}$. (c) A scatter correlation plot for *a*_1_ and $R_{0}$. (d) A scatter correlation plot for *a*_2_ and $R_{0}$.

**Fig 6:**
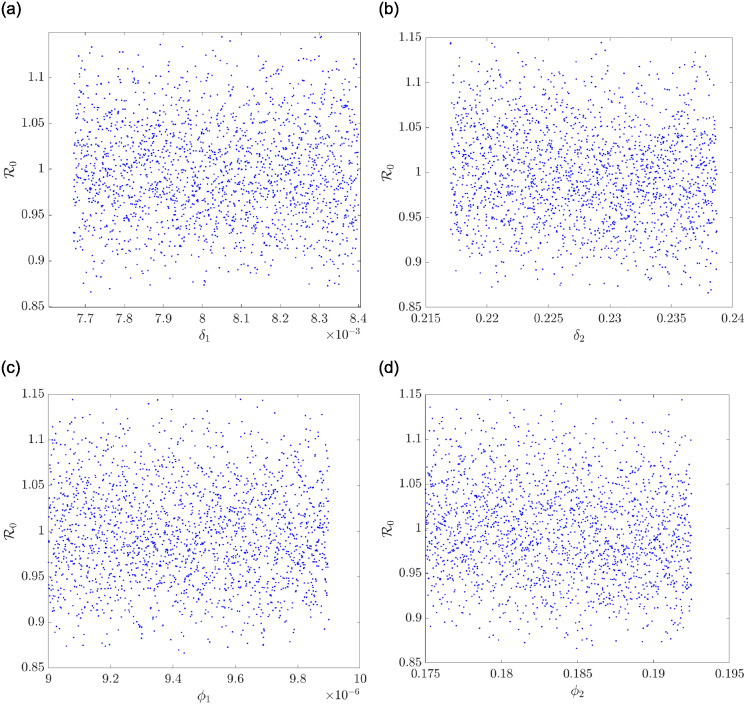
A scatter correlation plot for some parameters. (a) A scatter correlation plot for *δ*_1_ and $R_{0}$. (b): A scatter correlation plot for *δ*_2_ and $R_{0}$. (c): A scatter correlation plot for *ϕ*_1_ and $R_{0}$. (d) A scatter correlation plot for *ϕ*_2_ and $R_{0}$.

Now, in Figs 5 and 6, it is elaborated upon how some chosen parameters of the model correlate with the basic reproductive number of the co-dependence, $R_{0}$. Therefore, in Figs 5 and 6, a sample space of 2000 is used to establish the correlation. A positive correlation is observed in Fig 5a given the parameter *ξ*. This implies that a minor perturbation in *ξ* leads to a minor change in $R_{0}$ and in the same vein, a significant perturbation in *ξ* yields a significant response in *R*_0_. On the other hand, a negative correlation is observed in Fig 5b for the parameter *μ*; that is, a small perturbation of *μ* will result in a large change in $R_{0}$. In Fig 5a and 5d, it is observed that the parameters *a*_1_ and *a*_2_ correlate to $R_{0}$ positively, where this relationship is observed in *a*_1_ after 0.0048. In the same vein, we observe an increasing linear relationship between the parameters *δ*_1_, *δ*_2_, *ϕ*_1_ and *ϕ*_2_ and $R_{0}$ in Fig 6a to 6d.

## 5. Optimal control analysis of the drinking and smoking model

From Fig 4c, and based on the information in [43, 53–55], we develop an optimal control problem for model (1). The motive is to reduce the expansion level of smokers and drinkers and the co-dependence of the drinker and smoker populations. We incorporated five control variables that have a more significant impact on drastically controlling the number of people engaging in either drinking or smoking or both through mass campaigns against drinking and smoking using mainstream media, social media, educating the public, and instituting deliberate therapeutic, detoxification, counselling, and rehabilitation centres targeted at individuals involved in drinking or smoking or even both. The controls used are *u*_1_: drinking prevention efforts. *u*_2_: smoking prevention efforts. *u*_3_: recovery efforts on the co-dependence of drinking and smoking. *u*_4_: recovery efforts on drinking. *u*_5_: recovery efforts on tobacco. With these controls, the optimal control model equation is given as

$$\begin{array}{c} \begin{array}{cc} & \frac{dS}{dt}=\xi +\gamma_{1}R_{d}+\gamma_{2}R_{m}+\gamma_{3}R_{dm}-\left(1-u_{1}\right)\alpha_{1}S-\left(1-u_{2}\right)\alpha_{2}S-\mu S,\\ & \frac{dD}{dt}=\left(1-u_{1}\right)\alpha_{1}S-\left(\mu +\phi_{1}+\delta_{1}+u_{4}\right)D-\left(1-u_{2}\right)\alpha_{2}D,\\ & \frac{dM}{dt}=\left(1-u_{2}\right)\alpha_{2}S-\left(\mu +\phi_{2}+\delta_{2}+u_{5}+\left(1-u_{1}\right)\alpha_{1}\right)M,\\ & \frac{dDm}{dt}=\left(1-u_{2}\right)\alpha_{2}D+\left(1-u_{1}\right)\alpha_{1}M-\left(\mu +\phi_{3}+k_{1}+u_{3}\right)Dm,\\ & \frac{dR_{d}}{dt}=\left(\delta_{1}+u_{4}\right)D-\left(\mu +\gamma_{1}\right)R_{d},\\ & \frac{dR_{m}}{dt}=\left(\delta_{2}+u_{5}\right)M-\left(\mu +\gamma_{2}\right)R_{m},\\ & \frac{dR_{dm}}{dt}=\left(k_{1}+u_{3}\right)Dm-\left(\mu +\gamma_{3}\right)R_{dm}. \end{array}\} \end{array}$$ (19)

The controls *u*_1_, *u*_2_, and *u*_3_ are preventive control measures that are applied to prevent the susceptible class from becoming alcohol drinkers or tobacco smokers, or even both, as they are means of creating awareness of the dangers of the consumption of alcohol and tobacco. Also, *u*_4_ and *u*_5_ serve as treatment measures that are applied to the tobacco smoking and alcohol drinkers compartments. We then use the controls to minimise the control model, subject to the objective function defined as

$$\begin{array}{c} J=\int_{0}^{T}[A_{1}D+A_{2}M+A_{3}Dm+\frac{1}{2}w_{1}{u_{1}}^{2}+\frac{1}{2}w_{2}{u_{2}}^{2}+\frac{1}{2}w_{3}{u_{3}}^{2}+\frac{1}{2}w_{4}{u_{4}}^{2}+\frac{1}{2}w_{5}{u_{5}}^{2}]dt, \end{array}$$ (20)

where T is the final time. *A*_1_, *A*_2_, and *A*_3_ are the positive weight constants of the drinkers, smokers, and co-dependent populations of the drinkers and smokers, respectively. At the same time, $\frac{1}{2}w_{i}u_{i}^{2}$ is a positive coefficient that represents the costs of optimising the controls. We will determine the controls to minimise the population of smokers and drinkers and the costs incurred. $u_{1}^{⊛},u_{2}^{⊛},u_{3}^{⊛},u_{4}^{⊛}$ and $u_{5}^{⊛}$ in the form

$$\begin{array}{c} J\left(u_{1}^{⊛},u_{2}^{⊛},u_{3}^{⊛},u_{4}^{⊛},u_{5}^{⊛}\right)=\text{inf}\left\{J\left(u_{1},u_{2},u_{3},u_{4},u_{5}\right),\;\;u_{i}\epsilon U\right\}, \end{array}$$ (21)

where *U* = {(*u*_1_, *u*_2_, *u*_3_, *u*_4_, *u*_5_)/*u*_*i*_(*t*)} is Lebesgue measurable on [0, *T*], 0 ≤ *u*_*i*_ ≤ 1, *i* = 1, 2, 3, 4, 5} is a closed set.

### 5.1. The Hamiltonian and Optimality System

We formulate the Hamiltonian (H) by using Pontryagin’s maximum principle, as described in [56], which is defined as

$$\begin{array}{ccc} H & = & A_{1}D+A_{2}M+A_{3}Dm+\frac{1}{2}(w_{1}{u_{1}}^{2}+w_{2}{u_{2}}^{2}+w_{3}{u_{3}}^{2}+w_{4}{u_{4}}^{2}+w_{5}{u_{5}}^{2})\\ & & +\lambda_{1}(\xi +\gamma_{1}R_{d}+\gamma_{2}R_{m}+\gamma_{3}R_{dm}-\left(1-u_{1}\right)\alpha_{1}S-\left(1-u_{2}\right)\alpha_{2}S-\mu S)\\ & & +\lambda_{2}(\left(1-u_{1}\right)\alpha_{1}S-\left(\mu +\phi_{1}+\delta_{1}+u_{4}\right)D+\left(1-u_{2}\right)\alpha_{2}D)\\ & & +\lambda_{3}(\left(1-u_{2}\right)\alpha_{2}S-\left(\mu +\phi_{2}+\delta_{2}+u_{5}+\left(1-u_{1}\right)\alpha_{1}\right)M)\\ & & +\lambda_{4}(\left(1-u_{2}\right)\alpha_{2}D+\left(1-u_{1}\right)\alpha_{1}M-\left(\mu +\phi_{3}+k_{1}+u_{3}\right)Dm)\\ & & +\lambda_{5}(\left(\delta_{1}+u_{4}\right)D-\left(\mu +\gamma_{1}\right)R_{d})\\ & & +\lambda_{6}(\left(\delta_{2}+u_{5}\right)M-\left(\mu +\gamma_{2}\right)R_{m})+\lambda_{7}(\left(k_{1}+u_{3}\right)Dm-\left(\mu +\gamma_{3}\right)R_{dm}), \end{array}$$

with λ_*i*_, *i* = 1, 2, ⋯ been adjoint functions.

**Theorem 5.1**. *Let* $u_{1}^{⊛},u_{2}^{⊛},u_{3}^{⊛},u_{4}^{⊛},u_{5}^{⊛}$ *be an optimal control solutions that minimizes J over U, there are adjoint variable* λ_1_, ⋯, λ_7_ *such that*,

$$\begin{array}{cc} & \frac{d\lambda_{1}}{dt}=\lambda_{1}(\left(1-u_{1}\right)\alpha_{1}-\left(1-u_{2}\right)\alpha_{2}+\mu )-\lambda_{2}\left(1-u_{1}\right)\alpha_{1}+\lambda_{3}\left(1-u2\right)\alpha_{2},\\ & \frac{d\lambda_{2}}{dt}=-A_{1}+\lambda_{2}(\left(\mu +\phi_{1}+\delta_{1}+u_{4}\right)+\left(1-u_{2}\right)\alpha_{2})-\lambda_{4}\left(1-u_{2}\right)\alpha_{2}-\lambda_{5}\left(\delta_{1}+u4\right),\\ & \frac{d\lambda_{3}}{dt}=-A_{2}+\lambda_{3}((\mu +\phi_{2}+\delta_{2}+u_{5}+\left(1-u_{1}\right)\alpha_{1}))-\lambda_{4}\left(1-u_{1}\right)\alpha_{1}-\lambda_{6}\left(\delta_{2}+u_{5}\right),\\ & \frac{d\lambda_{4}}{dt}=-A_{3}+\lambda_{4}\left(\mu +\phi_{3}+k_{1}+u_{3}\right)-\lambda_{7}\left(k_{1}+u_{3}\right),\\ & \frac{d\lambda_{5}}{dt}=\lambda_{4}\left(\mu +\gamma_{1}\right)-\lambda_{1}\gamma_{1},\\ & \frac{d\lambda_{6}}{dt}=\lambda_{6}\left(\mu +\gamma_{2}\right)-\lambda_{1}\gamma_{2},\\ & \frac{d\lambda_{7}}{dt}=\lambda_{7}\left(\mu +\gamma_{2}\right)-\lambda_{1}\gamma_{3}, \end{array}$$

*and* λ_1_(*T*) = λ_2_(*T*) = λ_3_(*T*) = λ_4_(*T*) = λ_5_(*T*) = λ_6_(*T*) = λ_7_(*T*) = 0, *are the transversality conditions. The characterized control set is obtained as*

$$\begin{array}{cc} & u_{1}^{⊛}=\text{sup}\{0,\text{inf}(1,\frac{\lambda_{2}\alpha_{1}S+\lambda_{4}\alpha_{1}M-\lambda_{1}\alpha_{1}S}{w_{1}})\},\\ & u_{2}^{⊛}=\text{sup}\{0,\text{inf}(1,\frac{\lambda_{2}\alpha_{2}D+\lambda_{3}\alpha_{2}S+\lambda_{4}\alpha_{2}D-\lambda_{1}\alpha_{2}S}{w_{2}})\},\\ & u_{3}^{⊛}=\text{sup}\{0,\text{inf}(1,\frac{\lambda_{3}M+\lambda_{4}Dm-\lambda_{7}Dm}{w_{3}})\},\\ & u_{4}^{⊛}=\text{sup}\{0,\text{inf}(1,\frac{\lambda_{2}D-\lambda_{5}D}{w_{4}})\},\\ & u_{5}^{⊛}=\text{sup}\{0,\text{inf}(1,\frac{\lambda_{3}M-\lambda_{6}M}{w_{5}})\}. \end{array}$$

*Proof*. Using the Pontryagin’s maximum principle, we obtained the adjoint equations as follows

$$\begin{array}{cc} & \frac{d\lambda_{1}}{dt}=-\frac{dH}{dS}=\lambda_{1}(\left(1-u_{1}\right)\alpha_{1}-\left(1-u_{2}\right)\alpha_{2}+\mu )-\lambda_{2}\left(1-u_{1}\right)\alpha_{1}+\lambda_{3}\left(1-u_{2}\right)\alpha_{2},\\ & \frac{d\lambda_{2}}{dt}=-\frac{dH}{dD}=-A_{1}+\lambda_{2}(\left(\mu +\phi_{1}+\delta_{1}+u_{4}\right)-\left(1-u_{2}\right)\alpha_{2})-\lambda_{4}\left(1-u_{2}\right)\alpha_{2},\\ & \frac{d\lambda_{3}}{dt}=-\frac{dH}{dM}=-A_{2}+\lambda_{3}((\mu +\phi_{2}+\delta_{2}+u_{5}+k_{1}+u_{3}))-\lambda_{4}\left(1-u_{1}\right)\alpha_{1}-\lambda_{6}\left(\delta_{2}+u_{5}\right),\\ & \frac{d\lambda_{4}}{dt}=-\frac{dH}{dDm}=-A_{3}+\lambda_{4}\left(\mu +\phi_{3}+k_{1}+u_{3}\right)-\lambda_{7}\left(k_{1}+u_{3}\right),\\ & \frac{d\lambda_{5}}{dt}=-\frac{dH}{dR_{d}}=\lambda_{4}\left(\mu +\gamma_{1}\right)-\lambda_{1}\gamma_{1},\\ & \frac{d\lambda_{6}}{dt}=-\frac{dH}{dR_{m}}=\lambda_{6}\left(\mu +\gamma_{2}\right)-\lambda_{1}\gamma_{2},\\ & \frac{d\lambda_{7}}{dt}=-\frac{dH}{dR_{dm}}=\lambda_{7}\left(\mu +\gamma_{3}\right)-\lambda_{1}\gamma_{3}. \end{array}$$

Given the transversality conditions, λ_*i*_(*T*) = 0, *i* = 1, 2, ⋯. We used $\frac{\partial H}{\partial u_{i}}=0,i=1,2,\cdots$ that is partially differentiating the Hamiltonian equation with respect to *u*_1_, *u*_2_, *u*_3_, *u*_3_, *u*_4_, *u*_5_ to obtain the optimal controls; $u_{1}^{⊛},u_{2}^{⊛},u_{3}^{⊛},u_{3}^{⊛},u_{4}^{⊛},u_{5}^{⊛}$ as

$$\begin{array}{cc} & u_{1}^{⊛}=\frac{\lambda_{2}\alpha_{1}S+\lambda_{4}\alpha_{1}M-\lambda_{1}\alpha_{1}S}{w_{1}},\\ & u_{2}^{⊛}=\frac{\lambda_{2}\alpha_{2}D+\lambda_{3}\alpha_{2}S+\lambda_{4}\alpha_{2}D-\lambda_{1}\alpha_{2}S}{w_{2}},\\ & u_{3}^{⊛}=\frac{\lambda_{3}M+\lambda_{4}Dm-\lambda_{7}Dm}{w_{3}},\\ & u_{4}^{⊛}=\frac{\lambda_{2}D-\lambda_{5}D}{w_{4}},\\ & u_{5}^{⊛}=\frac{\lambda_{3}M-\lambda_{6}M}{w_{5}}. \end{array}$$

And the standard controls as follows

$$u_{1}^{⊛}=\{\begin{array}{cc} \Phi_{1}, & \;0<\Phi_{1}<1,\\ 0, & \;\Phi -1\leq 0,\\ 1, & \;\Phi_{1}\geq 1, \end{array}$$

$$u_{2}^{⊛}=\{\begin{array}{cc} \Phi_{2}, & \;0<\Phi_{2}<1,\\ 0, & \;\Phi_{2}\leq 0,\\ 1, & \;\Phi_{2}\geq 1, \end{array}$$

$$u_{3}^{⊛}=\{\begin{array}{cc} \Phi_{2}, & \;0<\Phi_{3}<1,\\ 0, & \;\Phi_{3}\leq 0,\\ 1, & \;\Phi_{3}\geq 1, \end{array}$$

$$u_{4}^{⊛}=\{\begin{array}{cc} \Phi_{2}, & \;0<\Phi_{4}<1,\\ 0, & \;\Phi_{4}\leq 0,\\ 1, & \;\Phi_{4}\geq 1, \end{array}$$

$$u_{5}^{⊛}=\{\begin{array}{cc} \Phi_{5}, & \;0<\Phi_{5}<1,\\ 0, & \;\Phi_{5}\leq 0,\\ 1, & \;\Phi_{5}\geq 1. \end{array}$$

In compacted form:

$$u_{1}^{⊛}=\text{sup}\left\{0,\text{inf}\left(1,\Phi_{1}\right)\right\},$$

$$u_{2}^{⊛}=\text{sup}\left\{0,\text{inf}\left(1,\Phi_{2}\right)\right\},$$

$$u_{3}^{⊛}=\text{sup}\left\{0,\text{inf}\left(1,\Phi_{3}\right)\right\},$$

$$u_{4}^{⊛}=\text{sup}\left\{0,\text{inf}\left(1,\Phi_{4}\right)\right\},$$

$$u_{5}^{⊛}=\text{sup}\left\{0,\text{inf}\left(1,\Phi_{5}\right)\right\},$$

where

$$\begin{array}{cc} & \Phi_{1}=\frac{\lambda_{2}\alpha_{1}S+\lambda_{4}\alpha_{1}M-\lambda_{1}\alpha_{1}S}{w_{1}},\\ & \Phi_{2}=\frac{\lambda_{2}\alpha_{2}D+\lambda_{3}\alpha_{2}S+\lambda_{4}\alpha_{2}D-\lambda_{1}\alpha_{2}S}{w_{2}},\\ & \Phi_{3}=\frac{\lambda_{3}M+\lambda_{4}Dm-\lambda_{7}Dm}{w_{3}},\\ & \Phi_{4}=\frac{\lambda_{2}D-\lambda_{5}D}{w_{4}},\\ & \Phi_{5}=\frac{\lambda_{3}M-\lambda_{6}M}{w_{5}}. \end{array}$$

The optimal control system is

$$\begin{array}{cc} & \frac{dS}{dt}=\xi +\gamma_{1}R_{d}+\gamma_{2}R_{m}+\gamma_{3}R_{dm}-\left(1-u_{1}\right)\alpha_{1}S-\left(1-u_{2}\right)\alpha_{2}S-\mu S,\\ & \frac{dD}{dt}=\left(1-u_{1}\right)\alpha_{1}S-\left(\mu +\phi_{1}+\delta_{1}+u_{4}\right)D-\left(1-u_{2}\right)\alpha_{2}D,\\ & \frac{dM}{dt}=\left(1-u_{2}\right)\alpha_{2}S-\left(\mu +\phi_{2}+\delta_{2}+u_{5}+\left(1-u_{1}\right)\alpha_{1}\right)M,\\ & \frac{dDm}{dt}=\left(1-u_{2}\right)\alpha_{2}D+\left(1-u_{1}\right)\alpha_{1}M-\left(\mu +\phi_{3}+k_{1}+u_{3}\right)Dm,\\ & \frac{dR_{d}}{dt}=\left(\delta_{1}+u_{4}\right)D-\left(\mu +\gamma_{1}\right)R_{d},\\ & \frac{dR_{m}}{dt}=\left(\delta_{2}+u_{5}\right)M-\left(\mu +\gamma_{2}\right)R_{m},\\ & \frac{dR_{dm}}{dt}=\left(k_{1}+u_{3}\right)Dm-\left(\mu +\gamma_{3}\right)R_{dm},\\ & \frac{d\lambda_{1}}{dt}=\lambda_{1}(\left(1-u_{1}\right)\alpha_{1}-\left(1-u_{2}\right)\alpha_{2}+\mu )-\lambda_{2}\left(1-u_{1}\right)\alpha_{1}+\lambda_{3}\left(1-u2\right)\alpha_{2},\\ & \frac{d\lambda_{2}}{dt}=-A_{1}+\lambda_{2}(\left(\mu +\phi_{1}+\delta_{1}+u_{4}\right)+\left(1-u_{2}\right)\alpha_{2})-\lambda_{4}\left(1-u_{2}\right)\alpha_{2}-\lambda_{5}\left(\delta_{1}+u4\right),\\ & \frac{d\lambda_{3}}{dt}=-A_{2}+\lambda_{3}((\mu +\phi_{2}+\delta_{2}+u_{5}+\left(1-u_{1}\right)\alpha_{1}))-\lambda_{4}\left(1-u_{1}\right)\alpha_{1}-\lambda_{6}\left(\delta_{2}+u_{5}\right),\\ & \frac{d\lambda_{4}}{dt}=-A_{3}+\lambda_{4}\left(\mu +\phi_{3}+k_{1}+u_{3}\right)-\lambda_{7}\left(k_{1}+u_{3}\right),\\ & \frac{d\lambda_{5}}{dt}=\lambda_{4}\left(\mu +\gamma_{1}\right)-\lambda_{1}\gamma_{1},\\ & \frac{d\lambda_{6}}{dt}=\lambda_{6}\left(\mu +\gamma_{2}\right)-\lambda_{1}\gamma_{2},\\ & \frac{d\lambda_{7}}{dt}=\lambda_{7}\left(\mu +\gamma_{2}\right)-\lambda_{1}\gamma_{3}, \end{array}$$

with the starting value conditions *S*(0) = *s*_0_, *D*(0) = *D*_0_, *M*(0) = *M*_0_, *Dm*(0) = *Dm*_0_, *R*_*d*_(0) = *R*_*d*0_, *R*_*m*_(0) = *R*_*m*0_, *R*_*dm*_ = *R*_*dm*0_ for λ_*i*_(*T*) = 0, *i* = 1, 2, ⋯. This completes the proof.

## 6. Numerical simulations

In this section, numerical simulations illustrate the dynamical patterns of the drinking and smoking models. We applied the Runge-Kutta fourth-order method to create a graphical resolution of the effect of contact rate and recovered rate on the co-dependence of drinkers and smokers. The parameter values used for the simulations are presented in Table 2. Using Matlab to show the effect of some parameters with and without optimal control strategies on drinking, smoking, and the co-dependence of drinking and smoking. The initial conditions used are *S*(0) = 0.6, *D*(0) = 0.2, *M*(0) = 0.1, *Dm*(0) = 0.06, *R*_*d*_(0) = 0.02, *R*_*m*_(0) = 0, *R*_*dm*_(0) = 0, with weight constants of *A*_1_ = 100, *A*_2_ = 100, *A*_3_ = 100, *w*_1_ = 0.5, *w*_2_ = 0.5, *w*_3_ = 0.5, *w*_4_ = 0.5, *w*_5_ = 0.5. We used the forward-backward sweep method using the Runge-Kutta fourth order.

### 6.1. Effect of control strategies and parameters on the model

**Fig 7:**
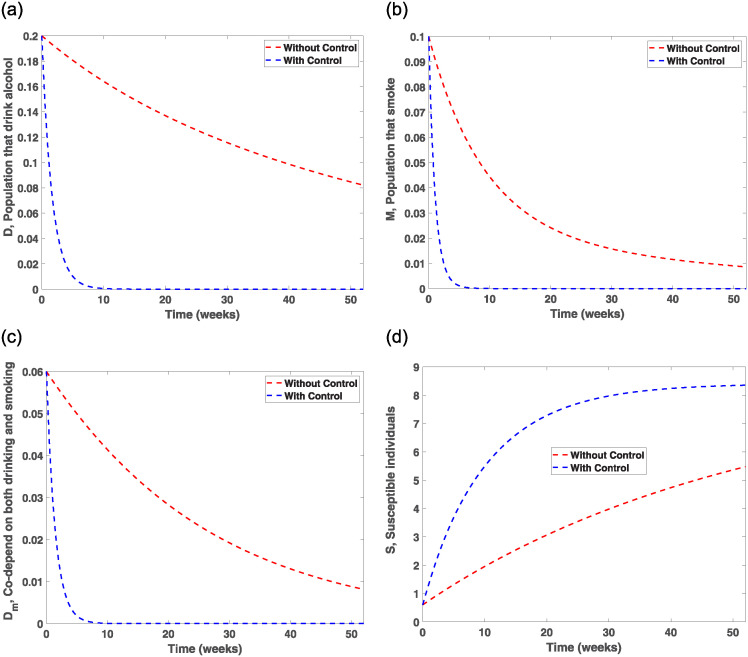
Sensitivity and optimal control trajectory plots. (a) An optimal control orbit of drinking only. (b) An optimal control orbit of smoking only. (c) An optimal control orbit of drinking and smoking co-dependence. (d) An optimal control orbit of Susceptible compartment.

**Fig 8:**
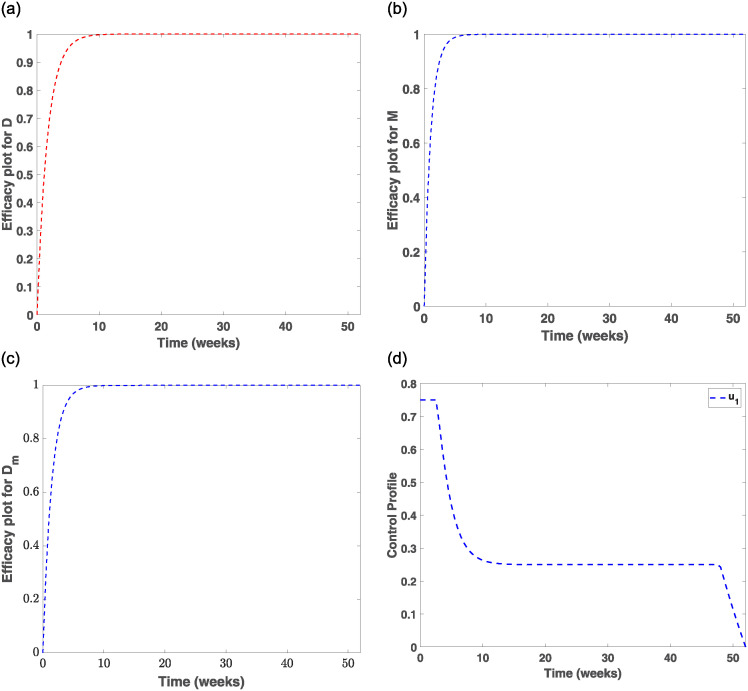
The control orbit plot for *u*_1_ and efficacy plots. (a) The efficiency of controls on drinkers compartment. (b) The efficiency of controls on smokers compartment. (c) The efficiency of controls on drinkers and smokers compartments. (d) The control orbit plot for *u*_1_.

**Fig 9:**
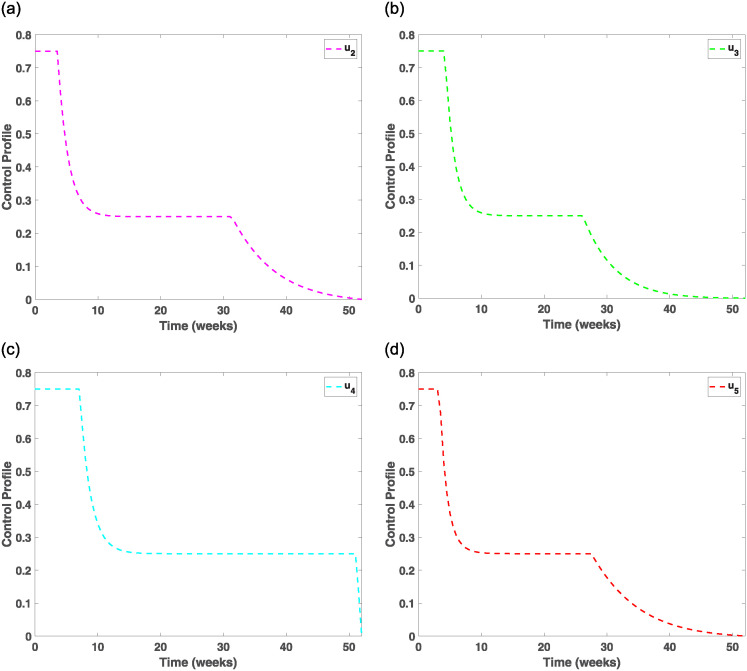
The control orbit plots for *u*_2_, *u*_3_, *u*_4_ and *u*_5_. (a) The control orbit plot for *u*_2_. (b) The control orbit plot for *u*_3_. (c) The control orbit plot for *u*_4_. (d) The control orbit plot for *u*_5_.

Evaluating the effects of treatment and prevention measures on smokers and drinkers, we investigated control strategies in the population for 52 weeks, which is recommended in the literature [42, 43]. Fig 7a indicates that prevention strategy I and treatment strategy IV are very effective control measures for drastically eliminating drinking behaviours among individuals. This observation is made as the number of individuals drinking starts to drop significantly from 0.2 in week one to 0.02 in week 4, then decreases slightly to zero from week 8 and beyond. This, therefore, indicates that drinking habits can be eliminated if advertisements of alcohol products are banned from mainstream media and drinkers are encouraged to patronise therapeutic sessions and detoxification services. To implement the control measure *u*_1_, Fig 8d shows that banning advertisement of alcohol products using the mainstream media should be enforced for the first 2 weeks at 0.75 and then stays stable from week 2 to week 48 before sharply dropping to zero in week 52, while Fig 9c shows that therapeutic and detoxification services should be set at 0.75 for the first 7 weeks and then drops and stabilises to 0.26 from week 10 to week 50, which then sharply decreases to almost zero in week 52. In addition, from Fig 8a, that is, the efficacy control plot, it is observed that it takes about 10 weeks for the control measures *u*_1_ and *u*_4_ to be approximately 100% effective in eradicating drinking behaviours from society.

**Fig 10:**
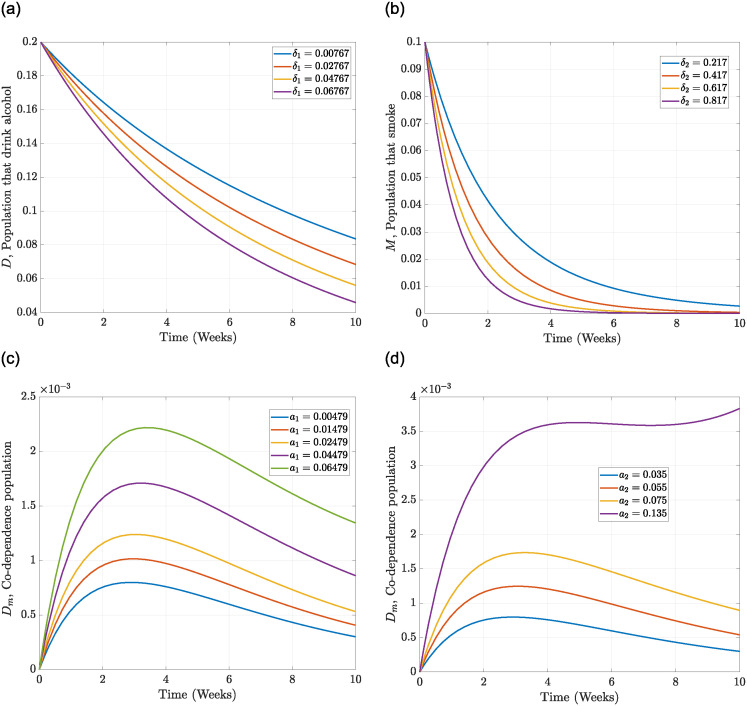
Parameter trajectory relation to the spread of drinking only, smoking only and drinking and smoking co-dependence. (a) the effect of an increasing *δ*_1_ trajectory on drinking only spread. (b) the effect of an increasing *δ*_2_ trajectory on smoking only spread. (c) the effect of an increasing *a*_1_ trajectory on drinking and smoking co-dependence spread. (d) the effect of an increasing *a*_2_ trajectory on drinking and smoking co-dependence spread.

Therefore, imposing high taxes on tobacco-related products and establishing counselling and rehabilitation centres to assist individuals in quitting smoking are highly effective measures compared to those without control, as evidenced by the sharp decrease in the number of smokers to zero during the first five weeks and after the simulation period. In Fig 10a, the control *u*_2_ is set at 0.75 for about three weeks and then decreases to 0.25 for the next 20 weeks before gradually reducing to zero in week 50. Fig 10d shows that the control strategy *u*_5_ will be appropriately applied if set at 0.75 for the first three weeks and then sharply decreased to 0.25 in week eight, stabilising from week 8 to week 28 before declining to zero in the 50th week. The efficacy of the controls (*u*_2_, *u*_5_) in Fig 8b shows that it takes about six weeks to obtain 100% efficiency.

Fig 7c indicates that if (*u*_1_, *u*_2_) are implemented concurrently, that is, banning advertisement of alcohol products through mainstream media, educating the public about the dangers associated with heavy drinking and smoking, and putting high taxes on tobacco products, it will take about eight weeks to drastically minimise the co-dependence on drinking and smoking as the control trajectory sharply decreases to 0.005 for the first four weeks and then gradually becomes asymptotic to zero from week eight and beyond. To further apply prevention strategy III, Fig 10b shows that the control should be set at 0.75 for the first four weeks and then drop to 0.26 in week ten and maintain a steady state for the next 17 weeks before it sharply decreases to zero in week 45. The efficacy of the control plot in Fig 8a reveals that it will take a maximum of 10 weeks to achieve approximately 100% effectiveness of the measures.

The efficiency of the control measures is demonstrated by Fig 7d, which illustrates how the number of susceptible persons increases abruptly for the first 20 weeks. The system then increases slightly and maintains a stable state during the simulation period when preventive methods and treatment measures are executed effectively. This, in essence, clarifies the reinfection incorporated into the model.

**Fig 11:**
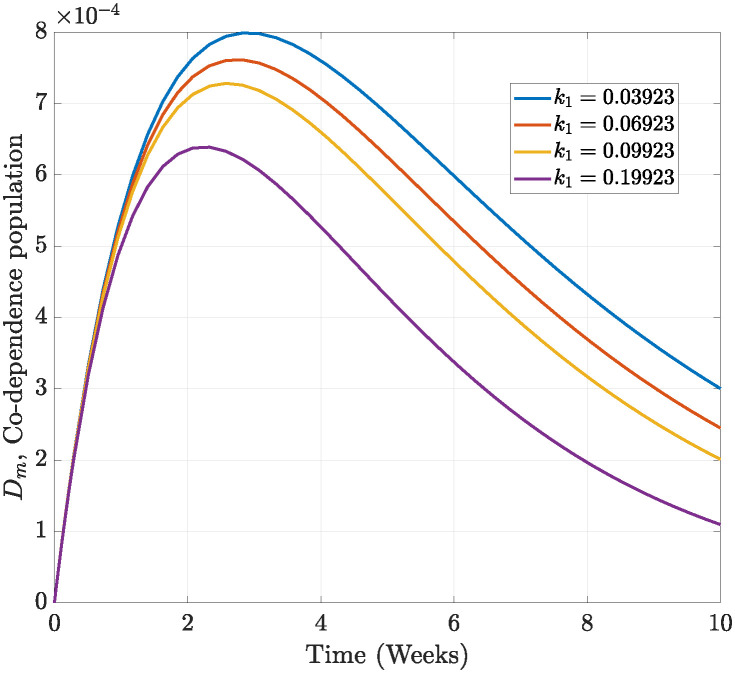
The effect of an increasing *k*_1_ trajectory on drinking and smoking co-dependence spread.

From Fig 10, we vary the value of the parameters *δ*_1_, *δ*_2_, *a*_1_, *a*_2_ and also *k*_1_ in Fig 11. We observe a decline in the number of individuals patronising alcoholic beverages as *δ*_1_; that is, the recovery rate of alcohol drinking increases in Fig 10a. A similar observation is made in Fig 10b, *δ*_2_; thus, the recovery rate of smoking is enhanced, and the number of individuals that smoke drastically reduces and is very close to eradication after five weeks. This indicates an inverse relationship, which consequently leads to a reduction in the basic reproductive number. This, therefore, implies that optimal control strategies that concentrate on individuals recovering from smoking and drinking only are very effective in minimising alcohol and tobacco consumption. On the other hand, a direct relationship is observed among the parameters *a*_1_, *a*_2_, *k*_1_ and the co-dependence on both alcohol drinking and smoking in Figs 10d to 11. In Fig 10c, it is observed that as *a*_1_ is increased, many individuals patronise both drinking and smoking, and a similar observation is made in Figs 10d and 11. Here, we observe that the basic reproductive number will rise whenever these parameters are increased. This further indicates that preventive measures are to be implemented to reduce the contact between the susceptible and the individuals infected with drinking and smoking.

## 7. Conclusion

A survey conducted by [9] reported that there exist the co-dependence on tobacco smoking and alcohol drinking in the population. Unfortunately, the existing literature concentrates on either the individual depending solely on alcohol or tobacco only whereas drinking and smoking have been known to be one of the leading causes of death globally as a result of their related effects on the body, causing severe health problems. For the certainty of controlling this socially unacceptable lifestyle, a mathematical model of the co-dynamics of drinking and smoking with optimal control is presented in this study. The model has been proven to be positive and bounded. The basic reproductive number ($R_{0}$) for drinking-free, smoking-free, and co-dependence of drinking and smoking-free equilibrium points were determined. It was established that the local and global stability of drinking-free and smoking-free equilibrium is asymptotically stable if their respective basic reproductive numbers, $R_{0D}$ and $R_{0M}$, are less than unity and unstable otherwise. Sensitivity analyses of the parameters were also performed, indicating that the contact and recovery rates significantly influence the spread and control of the co-dependence of drinking and smoking, thus considerably affecting their respective $R_{0}$. The study then suggested five control strategies, categorized under the headings prevention and treatment control strategies. Simulations of the controls were performed using the Forward-Backward Sweep Runge-Kutta method showed that the prevention controls measures like; public education, mass media campaigns, and implementing high taxes on alcohol and tobacco products—are very effective in reducing drinkers, smokers, and co-dependence populations. The simulations proved that the prevention strategies are productive in subsequently reducing the number of drinkers, smokers and the co-dependence of both anomalies. In addition, the treatment control strategies consisting of therapy, counselling, detoxification, and rehabilitation were observed to be very effective, thereby increasing the recovered drinkers, smokers, or co-dependence population. Therefore with no doubt, the current study reports that the treatment measures could drastically minimize the co-dependence population when applied effectively, thereby increasing the number of recovered individuals. The study further used numerical simulations to illustrate the dynamical behaviour of the model (co-dynamics of drinkers and smokers). We, therefore, encourage stakeholders to effectively implement the preventive and treatment control strategies outlined in this work. In the near future, this work will be extended by incorporating a passive smoking compartment and applying a fractal-fractional operator to study the continuous pattern and the effect of natural occurrences on this societal challenge.
